# An upgraded method of high-throughput chromosome conformation capture (Hi-C 3.0) in cotton (*Gossypium* spp.)

**DOI:** 10.3389/fpls.2023.1223591

**Published:** 2023-07-04

**Authors:** Jin Han, Siyuan Wang, Hongyu Wu, Ting Zhao, Xueying Guan, Lei Fang

**Affiliations:** ^1^ Zhejiang Provincial Key Laboratory of Crop Genetic Resources, The Advanced Seed Institute, Plant Precision Breeding Academy, College of Agriculture and Biotechnology, Zhejiang University, Hangzhou, China; ^2^ Hainan Institute of Zhejiang University, Yongyou Industry Park, Yazhou Bay Sci-Tech City, Sanya, China

**Keywords:** Hi-C, cross-linking, endonuclease fragmentation, chromatin interactions, chromatin loops, A/B compartments

## Abstract

High-throughput chromosome conformation capture (Hi-C) technology has been applied to explore the chromatin interactions and shed light on the biological functions of three-dimensional genomic features. However, it remains challenging to guarantee the high quality of Hi-C library in plants and hence the reliable capture of chromatin structures, especially loops, due to insufficient fragmentation and low efficiency of proximity ligations. To overcome these deficiencies, we optimized the parameters of the Hi-C protocol, principally the cross-linking agents and endonuclease fragmentation strategy. The double cross-linkers (FA+DSG) and double restriction enzymes (*Dpn*II+*Dde*I) were utilized. Thus, a systematic *in situ* Hi-C protocol was designed using plant tissues embedded with comprehensive quality controls to monitor the library construction. This upgraded method, termed Hi-C 3.0, was applied to cotton leaves for trial. In comparison with the conventional Hi-C 2.0, Hi-C 3.0 can obtain more than 50% valid contacts at a given sequencing depth to improve the signal-to-noise ratio. Hi-C 3.0 can furthermore enhance the capturing of loops almost as twice as that of Hi-C 2.0. In addition, Hi-C 3.0 showed higher efficiency of compartment detection and identified compartmentalization more accurately. In general, Hi-C 3.0 contributes to the advancement of the Hi-C method in plants by promoting its capability on decoding the chromatin organization.

## 1 Introduction

In the nuclei of multicellular eukaryotes, chromatin forms hierarchical three-dimensional (3D) structures on top of its linear conformation (Ouyang et al., 2020b). With the development of chromosome conformation capture (3C) methods (Louwers et al., 2009; Hakim and Misteli, 2012; Jamge et al., 2017; Hua et al., 2021), functional structures have been revealed at various genomic scales, including chromatin territories, A/B compartments, topologically associating domains (TADs), and chromatin loops (Meaburn and Misteli, 2007; Lieberman-Aiden et al., 2009; Dixon et al., 2012; Grob and Grossniklaus, 2017; Zheng et al., 2019). From territory to loop, the detection resolution required increases in order (Rodriguez-Granados et al., 2016).

The high-throughput chromosome conformation capture (Hi-C) method was developed in 2009 (Lieberman-Aiden et al., 2009), and greatly expanded the understanding of chromatin interactions and 3D genomics (Lesne et al., 2014; Schmitt et al., 2016; Szalaj and Plewczynski, 2018; Kong and Zhang, 2019). Hi-C technology has been improved continuously with optimizations decreasing random ligations and increasing signal-to-noise ratio. The initial dilution Hi-C (Hi-C 1.0) employed *Hind*III for chromatin fragmentation and conducted experimental reactions in lysed cells (Lieberman-Aiden et al., 2009). Subsequently, *Dpn*II replaced *Hind*III for endonuclease digestion and complete nuclei were isolated for *in situ* Hi-C (Kalhor et al., 2011; Rao et al., 2014). Hi-C 2.0 then integrated recent improvements and further optimized experimental parameters to develop a protocol that captured chromatin interactions at higher resolution (Belaghzal et al., 2017). Notably, the Micro-C method substituted micrococcal nuclease (MNase) for the restriction endonuclease enzyme and improved the resolution dramatically (Hsieh et al., 2015). In 2021, Dekker et al. systematically assessed Hi-C assays with distinct cross-linkers and fragmentation enzymes in human cells (Akgol Oksuz et al., 2021). The cross-linking chemistry included formaldehyde (FA), disuccinimidyl glutarate (DSG), and ethylene glycol-bis (EGS). The enzymes included *Hind*III, *Dpn*II, *Dde*I, and MNase. On this basis, a benchmarked Hi-C 3.0 protocol was proposed that combined the advantages of Hi-C 2.0 and Micro-C.

However, there are several technical barriers that still exist, such as the low resolution and high noise levels of Hi-C methods, heterogeneity of the experimental materials, and high cost due to the depth of sequencing (Ouyang et al., 2020b). As of yet, it’s still challenging to obtain a high-quality *in situ* Hi-C library, especially using plant samples. Solid cell walls and abundant secondary metabolites of plant tissues increase the difficulty of extracting intact nuclei (Tao et al., 2020), which hinders the acquisition of primary chromatin required for Hi-C library construction. Incomplete breaking of cell walls and the entry of cytoplasmic components into the digestive system can significantly interfere with chromatin fragmentation. Large and redundant genomes of many plants greatly raise the sequencing cost. Some crop genomes have a large number of repetitive sequences (Dong et al., 2017; Wang et al., 2017), making it difficult for Hi-C technology to achieve unique alignment on paired-end reads. Therefore, the rate of valid interactions is relatively low, varies from 20% to 48% and barely exceeds 50% (Wang et al., 2021; Pei et al., 2022; Yang et al., 2022). Therefore, there is a demand for the prompt development of an optimized Hi-C protocol in plants that can enhance data efficiency and increase the signal-to-noise ratio. Cotton (*Gossypium* spp.) is a representative crop with a polyploid genome and an abundant amount of gossypol on leaf, so cotton leaf was selected in the upgraded Hi-C method for trial.

Here, we applied double cross-linkers (FA+DSG) and double digestion enzymes (*Dpn*II+*Dde*I) to optimize the Hi-C protocol. This resulted in the first benchmarked Hi-C 3.0 workflow in plants. Nuclei acquisition and systematic quality controls were also incorporated to ensure the generation of a high-quality library. Compared to the conventional Hi-C 2.0, Hi-C 3.0 features major improvements in more reliable and stronger interaction signals, which contribute to the detection of chromatin loops and compartmentalization. Moreover, Hi-C 3.0 results in increased signal-to-noise ratio. This method provides a new option for investigating chromatin interactions and constructing high-quality Hi-C libraries in plants.

## 2 Materials and methods

### 2.1 Plant materials

Seedlings of cotton (*Gossypium hirsutum*) accession TM-1 (Texas Marker-1) were cultivated in an artificial light incubator with a photoperiod of 16 h (light)/8 h (dark), temperature of 28 ± 1°C, and 60 ± 5% humidity. The 4-5^th^ true leaves were sampled for Hi-C library construction, with one gram input for each library. Two biological replicates were applied for each library.

### 2.2 Reagents

#### 2.2.1 Enzymes

Biotin-14-dCTP (AAT Bioquest, 17019); dTTP (Sangon, B500050-0250); dATP (Sangon, B500044-0250); dGTP (Sangon, B500048-0250); DNA polymerase I, large (Klenow) fragment (NEB, M0210L); T4 DNA ligase (NEB, M0202S); proteinase K (NEB, P8107S); RNase A (Biosharp, BL543A); T4 DNA polymerase (NEB, M0203S); *Dpn*II (NEB, R0543S); *Dde*I (NEB, R0175S); NEBuffer 3 (NEB, B7003S).

#### 2.2.2 Kits

NEBNext Ultra II DNA Library Prep Kit (NEB, E7645L); VAHTS™ Multiplex Oligos set 4 for Illumina (Vazyme, N321).

#### 2.2.3 Chemicals

Potassium phosphate (K_3_PO_4_); sodium chloride (NaCl); sucrose; 37% Formaldehyde (Sigma-Aldrich, 252549); glycine; DSG Crosslinker (Leyan, 1134751); dimethyl sulfoxide (DMSO); 4-propanesulfonyl morpholine (MOPS); potassium chloride (KCl); ethylenediaminetetraacetic acid (EDTA); ethylene glycol tetraacetic acid (EGTA); spermidine (Macklin, S817735); spermine (Coolaber, CS10441); cOmplete™ EDTA-free Protease Inhibitor Cocktail (Roche, 11873580001); Tris-HCl; sodium hydroxide (NaOH); magnesium chloride (MgCl_2_); Triton X-100; Percoll (GE Healthcare, 17-0891-09); 1,4-dithiothreitol (DTT); sodium dodecyl sulfate (SDS); Tween-20; phenol:chloroform:isoamyl alcohol (25:24:1, v:v:v); sodium acetate (NaAc); isopropanol; ethanol; Streptavidin magnetic beads (NEB, S1420S); VAHTS DNA Clean Beads (Vazyme, N411-01).

### 2.3 Equipments

Miracloth (Millipore); centrifuge; Eppendorf microcentrifuge tubes; Magna GrIP™ Rack (Millipore); Bioruptor (Diagnode); PCR thermocycler; PCR strip tubes; agarose gel electrophoresis apparatus; Nanodrop apparatus.

### 2.4 Stock solutions (dissolved in double-distilled water, autoclaved prior to use)

1) 1 M K_3_PO_4_, pH 7.0: do not autoclave, 0.22 µm syringe filter unit (Millipore, SLGP033R) for sterilization
2) 1 M MOPS
3) 5 M NaCl
4) 1 M KCl
5) 1 M sucrose
6) 2 M sucrose
7) 0.3 M DSG: dissolved in DMSO (make a fresh stock of DSG in DMSO each time)
8) 2 M glycine
9) 1 M MgCl_2_
10) 20% (v/v) Triton X-100
11) 1 M Tris-HCl, pH 8.0: sodium hydroxide (NaOH) for pH adjustment
12) 10% (w/v) SDS: do not autoclave, 0.22 µm syringe filter unit (Millipore, SLGP033R) for sterilization
13) 0.5 M EDTA, pH8.0: sodium hydroxide (NaOH) for pH adjustment
14) 0.5 M EGTA, pH8.0: sodium hydroxide (NaOH) for pH adjustment
15) 1 M spermidine
16) 1 M spermine
17) 20% (v/v) Tween-20
18) 3 M NaAc, pH 5.2: HCl for pH adjustment

### 2.5 Working solutions (prepare fresh prior to use)

1) Cross-linking buffer 1: 10 mM K_3_PO_4_, pH 7.0; 50 mM NaCl; 0.4 M sucrose; 1% formaldehyde
2) Quench buffer 1: 10 mM K_3_PO_4_, pH 7.0; 50 mM NaCl; 0.4 M sucrose; 150 mM glycine
3) Cross-linking buffer 2: 10 mM K_3_PO_4_, pH 7.0; 50 mM NaCl; 0.4 M sucrose; 3 mM DSG
4) Quench buffer 2: 10 mM K_3_PO_4_, pH 7.0; 50 mM NaCl; 0.4 M sucrose; 400 mM glycine
5) Nuclei isolation buffer: 20 mM MOPS, pH 7.0; 40 mM NaCl; 90 mM KCl; 2 mM EDTA, pH 8.0; 0.5 mM EGTA, pH 8.0; 0.5 mM spermidine; 0.2 mM spermine; 1 × protease inhibitor cocktail (Nuclei isolation buffer without spermidine, spermine, and protease inhibitor cocktail can be stored at 4°C for months; prior to usage, add these three components freshly-prepared)
6) Sucrose-Percoll gradient centrifugation — Up buffer (SPGC-U buffer): 0.25 M sucrose; 10 mM Tris-HCl, pH 8.0; 10 mM MgCl_2_; 1% Triton X-100; 1 × protease inhibitor cocktail
7) Sucrose-Percoll gradient centrifugation — Down buffer (SPGC-D buffer): 1.7 M sucrose; 10 mM Tris-HCl, pH 8.0; 2 mM MgCl_2_; 0.1% Triton X-100; 1 × protease inhibitor cocktail
8) NEBuffer 3: 1 M NaCl; 500 mM Tris-HCl, pH 8.0; 100 mM MgCl_2_; 10 mM DTT
9) Blunt end ligation buffer (T4 DNA ligase reaction buffer): 300 mM Tris-HCl, pH 8.0; 100 mM MgCl_2_; 100 mM DTT; 1 mM ATP
10) SDS lysis buffer: 50 mM Tris-HCl, pH 8.0; 1% SDS; 10 mM EDTA, pH 8.0
11) TE buffer: 10 mM Tris-HCl, pH 8.0; 1 mM EDTA, pH 8.0
12) Tris elution buffer: 10 mM Tris-HCl, pH 8.0
13) TWB (Tween wash buffer): 5 mM Tris-HCl, pH 8.0; 0.5 mM EDTA, pH 8.0; 1 M NaCl; 0.05% (v/v) Tween-20
14) BB (Binding buffer): 10 mM Tris-HCl, pH 8.0; 1 mM EDTA, pH 8.0; 2 M NaCl

### 2.6 Protocol for *in situ* Hi-C 3.0

#### 2.6.1 Tissue fixation by double cross-linking

1) Cut 1 g fresh leaves into small pieces about 1 cm^2^ in size; immerse the leaves in 20 ml Cross-linking buffer 1 in a 50 ml tube. Vacuum infiltrate for 10 minutes at room temperature, then release the vacuum slowly.
2) Discard the Cross-linking buffer 1 and add 20 ml Quench buffer 1. Vacuum infiltrate for 5 minutes at room temperature to quench the fixation, then release the vacuum slowly.
3) Discard the Quench buffer 1 and rinse the leaves with ddH_2_O briefly.
4) Add 20 ml Cross-linking buffer 2 to the tube. Vacuum infiltrate for 10 minutes at room temperature twice, then release the vacuum slowly.
5) Discard the Cross-linking buffer 2 and add 20 ml Quench buffer 2. Vacuum infiltrate for 5 minutes at room temperature to quench the fixation, then release the vacuum slowly.
6) Discard the Quench buffer 2 and rinse the sample three times with ddH_2_O.
7) Dry the leaves between paper towels and press gently to absorb all liquid on the surface (see Note 1) in 2.6.5).

#### 2.6.2 Nuclei isolation and chromatin digestion (Day 1)

1) Prepare the Sucrose-Percoll gradient centrifugation buffer one hour prior to use. Mix 400 μl SPGC-U buffer and 600 μl Percoll to make 60% Percoll, then add 400 μl 60% Percoll to the bottom of a new 1.5 ml tube. Use a long pipette tip to transfer 200 μl SPGC-D buffer as the down layer carefully and slowly. Ensure there is a clear demarcation between the two layers. Put the tube on ice, maintaining the vertical orientation.
2) Grind the fixed samples to a fine powder in liquid nitrogen and transfer the powder to a 50 ml tube. Gently resuspend the powder with 25 ml ice-cold Nuclei isolation buffer.
3) Mix thoroughly and then filter the suspension through two layers of Miracloth into a new 50 ml tube on ice.
4) Centrifuge at 4°C, 1200 rcf for 10 min. Discard supernatant completely and quickly to avoid loosening the pellet. Use 2 ml ice-cold SPGC-U buffer to resuspend the pellet.
5) (Optional step) When extracting nuclei for the first time, it is necessary to estimate the total number of nuclei. Take 1 μl resuspended nuclei and stain with DAPI solution, then observe with a hemocytometer under a fluorescence microscope. A typical *in situ* Hi-C library construction requires 10^7^-10^8^ nuclei. This step should be done within 15 minutes, during which the remaining nuclei resuspension is kept on ice.
6) Transfer the resuspended nuclei gently to two 1.5 ml tubes, 1 ml per tube. Centrifuge at 4°C, 1200 rcf for 10 min and remove the supernatant.
7) Resuspend the pellet with 400 μl ice-cold SPGC-U buffer.
8) Load the resuspension on the top of the previously prepared Sucrose-Percoll gradient centrifugation tube. Centrifuge at 4°C, 1000 rcf for 15 min.
9) Remove the green-colored supernatant on the top. The brownish-white layer deposited on the interface is the nuclei fraction. Transfer this fraction carefully to a new 1.5 ml tube and combine nuclei from the same sample (separated at 2.6.2-6)).
10) Resuspend the pellet with 400 μl Nuclei isolation buffer. Centrifuge at 4°C, 500 rcf for 10 min and discard the supernatant.
11) Resuspend the pellet with 1 ml SPGC-U buffer. Centrifuge at 4°C, 1200 rcf for 5 min and discard the supernatant.
12) Repeat step 11) for one more wash. The pellet should be totally white and the supernatant transparent.
13) Gently resuspend the pellet with 300 μl 1 × NEBuffer 3 (dilute from 10 × to 1 × prior to use).
14) Centrifuge at 4°C, 3000 rcf for 5 min and discard the supernatant.
15) Gently resuspend the pellet with 150 μl 0.5% SDS; avoid producing bubbles. Aliquots 50 μl resuspension into three 2.0 ml tubes. Also transfer the remaining resuspension into a fourth tube to serve as a control without endonuclease enzyme treatment, and add 0.5% SDS to make a final volume of 50 μl.
16) Incubate samples at 62°C for 5 minutes to open up the chromatin.
17) Add 157.5 μl ddH_2_O and 12.5 μl 20% Triton X-100 to each tube to quench the SDS. Invert the tubes to mix well; avoid excessive foaming. Incubate samples at 37°C for 15 minutes.
18) Add 25 μl 10 × NEBuffer 3, 2.5 μl *Dpn*II (50 U) and 2.5 μl *Dde*I (50 U) into each sample tube. Add 30 μl NEBuffer 3 to the control tube. Invert the tubes to mix well. Incubate all tubes at 37°C overnight without shaking or rotating.

#### 2.6.3 Chromatin ligation (Day 2)

1) Incubate samples at 62°C for 20 minutes to deactivate the endonuclease enzymes, then cool to room temperature.
a. Quality control of digestion: Transfer 25 μl solution from each tube (including the control tube) to a new 1.5 ml tube. Add 50 μl ddH_2_O and 20 μl proteinase K to each tube. Incubate samples at 65°C for an hour. Add 100 μl phenol:chloroform:isoamyl alcohol (25:24:1, v:v:v) to each tube. Vortex vigorously for 30 seconds, then centrifuge at 12000 rcf for 5 min. Transfer 20 μl of the upper aqueous phase to a new 1.5 ml tube, then add 1 μl RNase A to the tube. Incubate at 37°C for 30 min. Examine DNA by electrophoresis on a 1% agarose gel. Compared to the undigested control chromatin, which exhibits a single bright band, the digested chromatin typically runs as a smear with a size range specific for the endonuclease enzymes applied.
b. Transfer the remainder of each solution to a new 2.0 ml tube, and add 25 μl 1 × NEBuffer 3 to each.
2) Add 1 μl each of 10 mM dTTP, dATP, dGTP and 25 μl 0.4 mM biotin-14-dCTP. Then add 14 μl ddH_2_O and 8 μl Klenow fragment (40 U) to each tube. Invert tubes gently to mix well. Incubate at 22°C for 4 h, inverting all tubes gently every 30 min.
3) Add 718 μl ddH_2_O, 120 μl blunt end 10 × ligation buffer, 50 μl 20% Triton X-100, and 5 μl T4 DNA ligase (2000 U) into each tube. Invert tubes gently to mix well. Incubate at 22°C for 4 h, inverting all tubes gently every 30 min.
4) Centrifuge at 22°C, 1000 rcf for 5 min and discard the supernatant. Resuspend the pellet with 750 μl SDS lysis buffer.
5) Add 10 μl proteinase K to each tube and incubate at 55°C for 30 min.
6) Add 30 μl 5 M NaCl to each tube and incubate at 65°C overnight to reverse the crosslinking.

#### 2.6.4 DNA purification, manipulation, and library amplification (Day 3)

1) Add 750 μl phenol:chloroform:isoamyl alcohol (25:24:1, v:v:v) to each tube. Vortex vigorously and centrifuge at 12000 rcf for 5 min. Transfer the upper aqueous phase to a new 2.0 ml tube. Then add 75 μl 3 M NaAc and 750 μl isopropanol to each tube. Invert to mix thoroughly.
2) Centrifuge at 4°C, 13000 rcf for 20 min and discard the supernatant. Wash the pellet with 80% ethanol.
3) Air dry the pellet, and then dissolve it in 100 μl TE buffer. Pipette up and down to completely dissolve.
4) Pool dissolved DNA from the same sample (separated at 2.6.2-15)). Add 1 μl RNase A to the tube. Incubate at 37°C for 30 min.
5) Add 1/10 volume of 3 M NaAc and an equal volume of isopropanol based on the combined sample volume. Invert and mix well.
6) Centrifuge at 4°C, 13000 rcf for 20 min and discard the supernatant. Wash the pellet with 80% ethanol, then air dry the pellet, and finally dissolve it with 55 μl Tris elution buffer.
a. Examine the DNA concentration by Nanodrop apparatus.
b. Quality control of ligation efficiency: Examine 5 μl DNA on a 1% agarose gel. Compared to the corresponding digestion control from 2.6.3-1)-a), successful proximity-ligated chimeras should have a higher molecular weight (see Note 2) in 2.6.5).
7) Add 10 μl T4 DNA polymerase buffer (NEBuffer 2.1), 1 μl 10mM dGTP, 1 μl 10 mM dATP, 3 μl T4 DNA polymerase (10 U), and 35 μl ddH_2_O to 50 μl of recovered DNA. Mix well and incubate at 20°C for 4 h.
8) Add 2 μl 0.5 M EDTA, pH 8.0 to each tube to stop the reaction.
9) Add 28 μl ddH_2_O to each tube to yield a final volume of 130 μl.
10) Transfer sample to tubes suitable for sonication.
a. Quality control of sonication: Aliquot 10 μl sample as sonication control and keep it at 4 °C for temporary storage. Sonicate the remaining sample with a Bioruptor (Diagnode) at 4 °C using 30 s on/30 s off per cycle, 8 cycles per round, invert and spin briefly after each round. Load 10 μl sonicated DNA and the control on a 1.5% agarose gel to check the effect of sonication. Sonicate the DNA to a smear size ranging around 300-500 bp (which will require three or more rounds in total).
b. Transfer 100 μl sheared DNA to a new 1.5 ml tube.
11) Add 80 μl (0.8 × sample volume) of resuspended VAHTS DNA Clean Beads. Pipette up and down several times to mix well. Incubate at room temperature for 5 min.
12) Place the tube on a magnetic separation stand, and discard the supernatant carefully when the solution is clear.
13) Keep the tube on the magnetic separation stand, and add 1 ml freshly prepared 80% ethanol to the tube without disturbing the beads. Incubate at room temperature for 30 sec. Discard the supernatant carefully. Repeat rinse once.
14) Briefly spin the tube and then put it back on the magnetic separation stand. Remove the remaining ethanol completely and air dry the tube for 5-10 min with the lid open, still on the separation stand.
15) Elute target DNA from the beads with 310 μl nuclease-free water. Pipette up and down to mix well. Put the tube on the magnetic separation stand and wait until the solution is all clear. Transfer 300 μl supernatant to a new 1.5 ml tube.
16) Prepare streptavidin magnetic beads for pulldown of biotinylated ligation products. Quantify the DNA in each library by Nanodrop apparatus to determine the amount of beads needed for pulldown. Use 2 μl beads per 1 μg DNA input, with a minimum of 10 μl beads. Vortex gently to mix the beads well and transfer an appropriate volume to a new 1.5 ml tube.
17) Wash the beads with 400 μl TWB by pipetting. Incubate at room temperature for 3 min with rotation. Capture the beads on a magnetic separation stand for 1 min and discard the supernatant.
18) Resuspend the beads with 300 μl BB and transfer them to the tube with supernatant from 2.6.4-15). Incubate at room temperature for 15 min with rotation. Capture the beads on a magnetic separation stand for 1 min and discard the supernatant.
19) Resuspend the beads with 600 μl TWB and transfer to a new 1.5 ml tube. Capture the beads on a magnetic separation stand and discard the supernatant. Repeat rinse once.
20) Resuspend the beads with 100 μl Tris elution buffer. Transfer the resuspended beads to a new 200 μl tube. Capture the beads on a magnetic separation stand and discard the supernatant.
21) Resuspend the beads with 50 μl Tris elution buffer.
22) End repair, dA-tailing
Add the following reagents to the 200 μl tube:
NEBNext Ultra II End Prep Enzyme Mix, 3 μl;
NEBNext Ultra II End Prep Reaction Buffer, 7 μl;
23) Pipette up and down several times to mix completely. Spin briefly to collect all the liquid.
24) Incubate at 20°C for 30 min with heat lid off.
25) Incubate at 65°C for 30 min with heat lid set to 80°C; pipette up and down several times to mix completely every 10 min.
26) Ligation reaction
Add the following reagents to the 200 μl tube in the order given:
Adaptor (5 μM), 2.5 μl (see Note 3) in 2.6.5);
NEBNext Ligation Enhancer, 1 μl;
NEBNext Ultra II Ligation Master Mix, 30 μl (mix by pipetting up and down several times prior to adding to the reaction)
27) Pipette the entire volume up and down at least ten times to mix thoroughly. Perform a quick spin to collect all liquid from the sides of the tube.
28) Incubate at 20°C for 15 min with heat lid off.
29) Place the tube on a magnetic separation stand to separate the beads from the supernatant.
30) Resuspend the beads with 100 μl TWB, transfer the liquid to a new 1.5 ml tube, and then add another 500 μl TWB. Reclaim the beads on a magnetic separation stand and discard the supernatant. Repeat rinse once.
31) Resuspend the beads with 400 μl Tris elution buffer. Transfer the resuspended beads to a new 1.5 ml tube. Reclaim the beads on a magnetic separation stand and discard the supernatant.
32) Resuspend the beads with 250 μl Tris elution buffer.
33) Library preparation
Add the following reagents to a PCR tube for amplification:
Beads (DNA fragments), 16 μl;
NEBNext Ultra II Q5 Master Mix, 20 μl;
i5 primer (10 μM), 2 μl
i7 primer (10 μM), 2 μl
34) Titration PCR amplification
PCR protocol is as follows (see Note 4**)** in 2.6.5):
30 seconds at 98°C
10 (more or less) cycles of:
10 seconds at 98°C
75 seconds at 65°C
5 minutes at 65°C
35) After the PCR amplification, bring the total volume of the library to 55 μl with ddH_2_O.
36) Separate beads on a magnetic separation stand. Transfer 50 μl of the supernatant to a new 1.5 ml tube. Transfer 2 μl of the remaining sample to another tube and put it on ice, as control for final library quality.
37) Add 35 μl (0.7 × sample volume) of resuspended VAHTS DNA Clean Beads to the tube. Pipette up and down several times to mix well. Incubate at room temperature for 5 min.
38) Place the tube on a magnetic separation stand, and discard the supernatant carefully when the solution is clear.
39) Keeping the tube on the magnetic separation stand, add 1 ml freshly prepared 80% ethanol to the tube without disturbing the beads. Incubate at room temperature for 30 sec. Discard the supernatant carefully. Repeat rinse once.
40) Resuspend the beads with 40 μl nuclease-free water. Add 28 μl (0.7 × sample volume) of resuspended VAHTS DNA Clean Beads to the tube. Pipette up and down several times to mix well. Incubate at room temperature for 5 min.
41) Place the tube on a magnetic separation stand, and discard the supernatant carefully when the solution is clear.
42) Keeping the tube on magnetic separation stand, add 1 ml freshly prepared 80% ethanol to the tube without disturbing the beads. Incubate at room temperature for 30 sec. Discard the supernatant carefully. Repeat rinse once.
43) Briefly spin the tube and then put it back on the magnetic separation stand. Remove the remaining ethanol completely and air dry the tube for 5-10 min with the lid open while on the magnetic separation stand.
44) Elute target DNA from the beads with 20 μl nuclease-free water. Pipette up and down to mix well. Put the tube on the magnetic separation stand and wait until the solution is all clear. Transfer 17 μl supernatant to a new 1.5 ml tube and store at -80°C for high-throughput sequencing. Use 2 μl of the remaining sample to check the size selection and DNA purification efficiency by running a 1.5% agarose gel, comparing against the control from 2.6.4-36).
45) Sequence the library on a NovaSeq platform with 150 bp paired-end reads (PE150).

#### 2.6.5 Notes

1) The fixed samples can be flash-frozen in liquid nitrogen and stored at -80°C for a long time. Once the stored samples are thawed, it is recommended to proceed through all remaining steps in order to avoid repeated freezing and thawing.
2) The recovered DNA can be stored at -20°C for an extended period. However, it is recommended to immediately continue with the following DNA treatments and library construction.
3) Adaptor is from the VAHTS™ Multiplex Oligos set 4 for Illumina (Vazyme, N321), as are the i5 and i7 primers.
4) To select the most appropriate number of cycles for PCR amplification, the rule of thumb is to use the lowest number of cycles that can yield a visible smear on a gel.

### 2.7 Data analysis

#### 2.7.1 Sequencing strategy and data quality evaluation

Parallel libraries were constructed with the Hi-C 2.0 and Hi-C 3.0 methods from the same plant materials. Every library was sequenced to acquire a small amount of data (~15-20 G) for pilot testing the sequencing quality, read-mapping rates and valid interaction rates. Based on the assessments of the obtained libraries from the pilot test, the total data required for a high-quality Hi-C library could be estimated with respect to the valid interaction rate, the resolution level of interest and the plant genome size. Here, the target data size of every library was about 200 giga base pairs (Gb). Sequencing and data analysis service was provided by Wuhan Frasergen Bioinformatics Co. Ltd.

Adapters and low-quality reads were filtered from the raw reads to yield clean data using trimmomatics (Version: 0.39). Further analysis was based on the clean data here after with FastQC checking the data quality.

#### 2.7.2 Reproducibility analysis and Hi-C data mapping

The concordance of the four libraries was assessed via GenomeDISCO (Ursu et al., 2018) (integrated by 3DChromatin_ReplicateQC, http://github.com/kundajelab/3DChromatin_ReplicateQC). And the replicates from the same group having high correlations were combined for subsequent analysis to increase the resolution of the Hi-C interaction heatmap.

The conventional Hi-C 2.0 method used HiC-Pro (Servant et al., 2015) for data processing, but Hi-C 3.0 involves fragmenting with two enzymes and hence a diversity of ligation events. Thus, the strategy of separation in the junction point and paired mapping employed in the HiC-Pro pipeline is not suitable for Hi-C 3.0. Instead, the compatible program distiller (https://github.com/mirnylab/distiller-nf) was adjusted for processing of both Hi-C 2.0 and Hi-C 3.0 data. Firstly, the clean paired-end reads were mapped to the reference genome using bwa mem -SP. Next, the pairtools software (https://github.com/mirnylab/pairtools) was applied, with pairtools parse used to convert alignments to.pair format, pairtools sort (pairtools version: 0.3.0) to sort reads, and pairtools dedup for PCR duplication removal to yield valid pairs.

#### 2.7.3 *Cis* and *trans* ratios

Pairtools provided statistics regarding the number of interactions captured within and between chromosomes, respectively intra-chromosomal (*cis*) and inter-chromosomal (*trans*) interactions. The ratio of *cis* or *trans* interactions was calculated by dividing the total number of interactions of that type by the count of all valid interactions. Distance-separated *cis* interactions were likewise calculated by dividing the total *cis* interactions occurring within a certain interval by the count of all *cis* interactions.

#### 2.7.4 Matrix construction, adjustment, and decay curve

The software cooler (https://github.com/mirnylab/cooler.git) was employed to generate interaction maps of the valid pairs. First, cooler cload (cooler version: 0.8.11) was applied to convert from. pair to. cool format. Next, cooler balance was utilized for the balance and adjustment of contact matrices. Downstream analysis all used.cool files as input. Additionally, cooler was used to calculate and pairsqc was used to construct the decay curve of the average contact probability with increased interaction distance, known as the *P*(s) plot, the max_logdistance was set to be log10(longest chromosome).

#### 2.7.5 Identification of compartments

At a resolution of 100 kb, the cool tools eigs-cis utility was applied to detect compartments. First, gene density was integrated to identify compartments (Imakaev et al., 2012), and then those compartments were assigned based on the profiles such that A compartments have a high gene density and B compartments a low gene density.

Next, cool tools saddle and principal component analysis results (Lieberman-Aiden et al., 2009) were combined to integrate and quantify A-A and B-B compartment interactions. The interaction matrix was sorted based on the eigenvector values from lowest to highest (B to A). Sorted maps were then normalized for their expected interaction frequencies to generate the saddle plots. To quantify interactions, the strongest 20% of A–A interactions and the strongest 20% of B–B interactions were normalized by the bottom 20% of A–B interactions. The formula is as follows: y = top(B–B)/bottom(A–B) and x = top(A–A)/bottom(A–B) (Akgol Oksuz et al., 2021).

#### 2.7.6 Identification of TADs

At a resolution of 40 kb, the hicexplorer sub-tool hicFindTAD was adopted to detect TADs. Regions were binned according to insulation score (Crane et al., 2015), and then interactions of regions upstream or downstream of each bin were identified at the whole-genome level. Afterwards, the extreme low points of the insulation score curve were determined. These points corresponded to the TAD boundaries, of which weak boundaries were filtered by hicFindTAD (–correctForMultipleTesting fdr –thresholdComparisons 0.01 –delta 0.01).

#### 2.7.7 Identification of loops

At a resolution of 10 kb, the Fit-Hi-C (v2.0.8) with parameter -p 2 was used to evaluate interactions within and between the chromosomes of each sample (Ay et al., 2014). Corresponding *p-*values and *q*-values were calculated between contact bins across the whole genome, and an interaction was determined to be significant when the *p*-value and *q*-value were both less than 0.01 and the number of contact reads was more than 2. Significant intra-chromosomal interactions between two non-adjacent bins were considered *cis* loops, while significant inter-chromosomal interactions were considered *trans* loops. Genome-wide *cis* loops were sorted from small to large on *p*-value, while *trans* loops were sorted from large to small on the number of contact reads supporting the interaction.

## 3 Results

### 3.1 An optimized *in situ* Hi-C 3.0 method for plants

**Figure 1:**
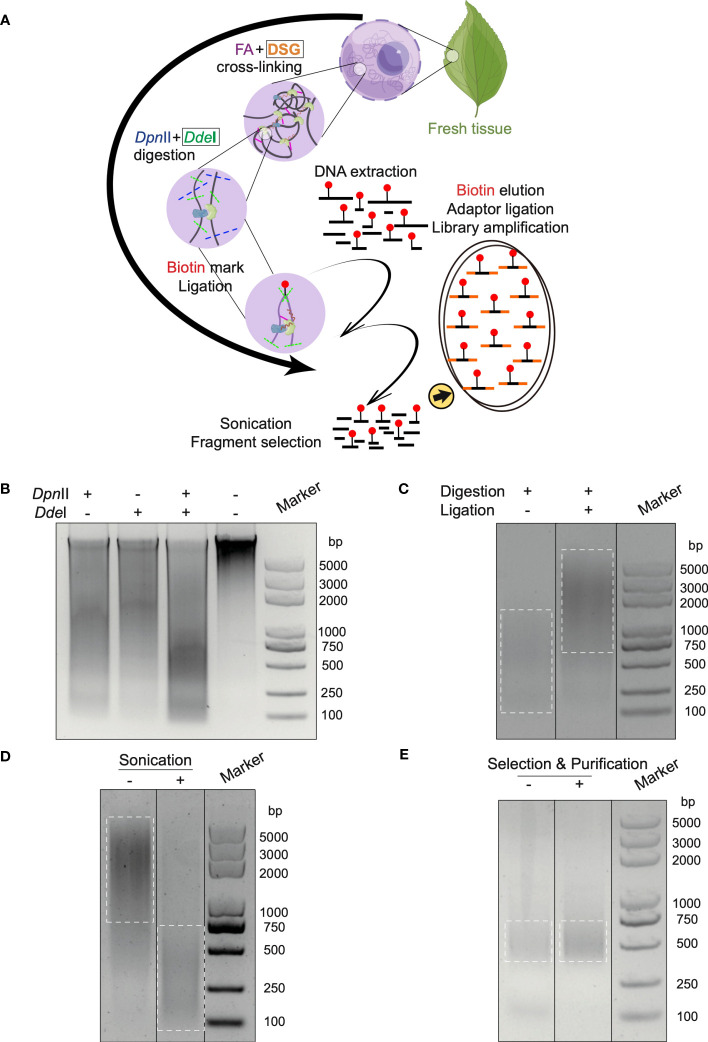
*In situ* Hi-C 3.0 method for plants with quality controls. **(A)** Schematic workflow of the Hi-C 3.0 protocol in plants. 1) Cross-linking is conducted using fresh tissues with FA and DSG. 2) Adjacent chromatin segments are fixed *in situ*. 3) Nuclei are isolated and chromatin is digested by *Dpn*II and *Dde*I. 4) Overhangs are filled with nucleotides, one of which is biotinylated (red dot). 5) Proximity ligation results in chimeras. 6) The cross-linking is reversed, and DNA is extracted and purified. 7) DNA fragments are sonicated and selected. 8) Biotinylated ligation products are pulled down and used for library construction. Black boxes indicate the differences between Hi-C 2.0 and 3.0. **(B-E)** Quality control of key steps in the Hi-C 3.0 protocol. **(B)** DNA digested by *Dpn*II and/or *Dde*I, and intact primary genomic DNA. **(C)** Adjacent DNA fragments ligated by T4 DNA ligase. **(D)** DNA fragmentation to size of ~200-500 bp by sonication. **(E)** Evaluation of the final Hi-C 3.0 library before or after fragment selection and purification.

According to the continuous development of 3C-derived methods (Han et al., 2018) and Hi-C based technologies (Kempfer and Pombo, 2020), cross-linking and chromatin fragmentation as two critical factors are distilled (**Supplementary Table S1**) (Lieberman-Aiden et al., 2009; Belaghzal et al., 2017; Hsieh et al., 2015; Hsieh et al., 2016; Akgol Oksuz et al., 2021). In Hi-C 3.0, nuclear chromatin was fixed using double cross-linking agents: FA for proximity linkage and DSG for long-distance linkage. This differs from Hi-C 2.0, which used only FA for fixation. The chromatin was digested with double endonuclease enzymes of *Dpn*II and *Dde*I, instead of single enzyme in Hi-C 2.0, to generate fine DNA fragmentation (**Figure 1A**). In addition, the purification step after nucleus isolation was added to maintain an intact ambient condition for the experimental reactions, which is described in the method section.

### 3.2 Systematic quality control of critical Hi-C procedures

The *in situ* Hi-C assay involves a long process and lacks technique controls to ensure the effective performance of critical procedures such as chromatin fragmentation, ligation, and library amplification. It is essential to apply the quality controls to guarantee a precise procedure and a high-quality library. Therefore, a quality control system was integrated into the protocol to monitor key experimental products by agarose gel electrophoresis.

First, the chromatin was digested by restriction endonuclease enzymes. Gel electrophoresis confirmed the primary DNA to be clear and intact before digestion, while digested DNA showed a smear (**Figure 1B**). Notably, DNA digested by both *Dpn*II and *Dde*I formed a smaller smear enriched in the 200-750 bp range, in contrast with single digestion by *Dpn*II (500-2000 bp) or *Dde*I (1500-2000 bp) respectively (**Figure 1B**). Second, the ligation reaction was the next most significant step. The chromatin segments after ligation exhibited increased molecular weight compared to the un-ligated DNA (**Figure 1C**). Third, sonication sheared the DNA into smaller sizes (< 500 bp) for Illumina library construction (**Figure 1D**). Last, gel electrophoresis confirmed the Hi-C library to comprise a smear of fragments around 300-500 bp after amplification (**Figure 1E**). After the removement of primer dimers, the final library was enriched with DNA fragments at size of 300-500 bp after selection and purification (**Figure 1E**).

The above controls were also performed at the corresponding steps in Hi-C 2.0 protocol (**Supplementary Figure S1A-D**). Additionally, the new restriction site created through ligation of the blunted ends digested by *Dpn*II in the final library underwent *Cla*I digestion (**Supplementary Figure S1E**).

### 3.3 Hi-C 3.0 improves data efficiency and increases signal-to-noise ratio

**Figure 2:**
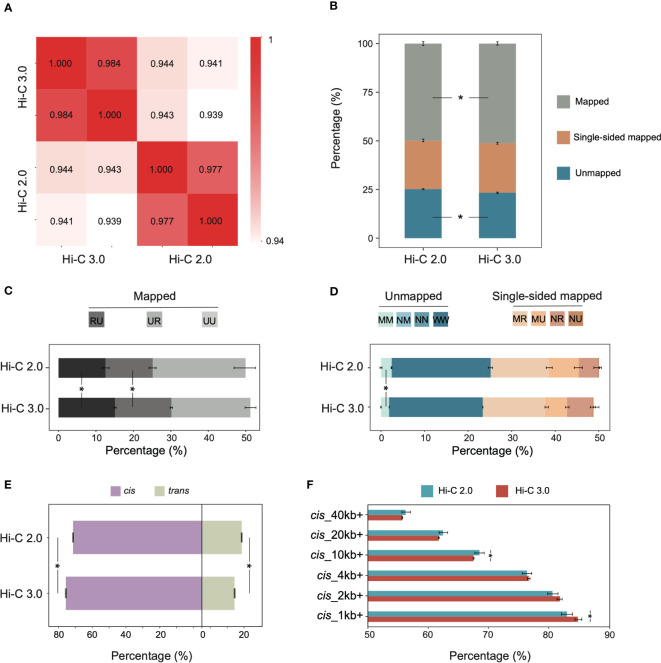
Hi-C 3.0 improves data efficiency and increases signal-to-noise ratio. **(A)** Reproducibility assessment of Hi-C libraries, Hi-C 2.0 and 3.0 each with two biological replicates. **(B)** The distribution of mapped reads, single-sided mapped and unmapped reads for Hi-C 2.0 and 3.0. Mapped reads indicated valid data, single-sided mapped and unmapped reads constituted invalid data. **(C)** The percentage of distinct mapped reads to total reads after identification of ligation junctions. Codes UU, UR, and RU represent mapped read pairs. **(D)** The percentage of distinct single-sided mapped and unmapped reads to total reads after identification of ligation junctions. Codes NU (null-unique), NR (null-rescued), MU (multi-unique), MR (multi-rescued) represent single-sided mapped read pairs, while MM (multi-multi), NM (null-multi), NN (null-null), and WW (walk-walk) represent unmapped read pairs. **(E)** The percentage of *cis*/*trans* read pairs to valid read pairs. **(F)** The percentage of *cis* read pairs with different interaction ranges to total *cis* read pairs. Bars indicate mean ± SD for two biological replicates. Significance determined by Student’s *t*-test. *P < 0.05.

To compare Hi-C 2.0 and the upgraded Hi-C 3.0, we generated libraries from the same cotton leaf tissue using both protocols. Each group had two biological replicates, and each individual library produced ~600-800 million clean read pairs (**Supplementary Table S2**). The concordance of contact maps showed that libraries from the same group exhibited the highest reproducibility (**Figure 2A**). Accordingly, reads from two biological replicates for each method were combined for subsequent analyses to increase the resolution of interaction matrix (Crane et al., 2015; Hug et al., 2017).

The HiC-Pro pipeline is commonly used for processing Hi-C data, and it has validated the high quality of the Hi-C 2.0 data used in this study (**Supplementary Figure S2**) (Servant et al., 2015). While HiC-Pro cannot be applied to Hi-C 3.0 due to the complicated ligation junctions induced by two digestion enzymes. Therefore, the software Pairtools was adapted for both Hi-C 2.0 and 3.0 data analysis, and depicted the validity of reads by category. The clean reads were aligned to the reference genome of cotton TM-1 (v2.1) (Hu et al., 2019). Compared to Hi-C 2.0, Hi-C 3.0 produced less unmapped reads and more mapped reads, especially ones with uniquely aligned pair-ends (**Figure 2B**). Intriguingly, the valid data rate of Hi-C 3.0 (51.21%) was rather high for plant Hi-C samples, which varied from 17% to 45% (**Supplementary Table S3**) (Yang et al., 2022; Dong et al., 2020; Ricci et al., 2019; Wang et al., 2021; Wang et al., 2017; Pei et al., 2022). Alignment of the paired-end reads was represented by two letters of U (Unique), R (Rescue), N (Null), M (Multi), or W (Walk). The mapped reads coded as UU, UR, or RU constituted valid pairs (**Supplementary Figure S3A**). Both single-end mapped reads coded as NU, NR, MU or MR and unmapped reads coded as WW, NN, NM, or MM represented invalid pairs (**Supplementary Figure S3B**). Notably, Hi-C 3.0 increased the ratio of valid pairs by raising the fraction of RU and UR (**Figure 2C**), and decreased the ratio of invalid pairs by reducing the fraction of MM, WW, and MU (**Figure 2D**).

Given that each chromosome occupies its own territory, the true interactions often occur within chromosomes (Schmitt et al., 2016). Hence, the ratio of intra-chromosomal/inter-chromosomal (*cis*/*trans*) contacts usually serves as a quality indicator for Hi-C library (Lajoie et al., 2015). Hi-C 3.0 increased the *cis* proportion to 80.7% and reduced the *trans* proportion to 19.3%, and significantly elevated the *cis*/*trans* ratio (**Figure 2E**). The *cis* proportion was a great improvement compared to the previous Hi-C data in plants (**Supplementary Table S3**). Among *cis* interactions, Hi-C 3.0 generated more contacts between loci separated by less than 10 kb than did Hi-C 2.0, which resembled the Micro-C (Hsieh et al., 2015). Meanwhile, the contacts involving longer distances (> 20 kb) did not show an obvious decline (**Figure 2F**). These data indicated that the Hi-C 3.0 protocol improves the efficiency of valid data and obtains more short-range *cis* signals than *trans* interactions without losing some long-range interactions.

### 3.4 Hi-C 3.0 detects more contacts at higher resolution

**Figure 3:**
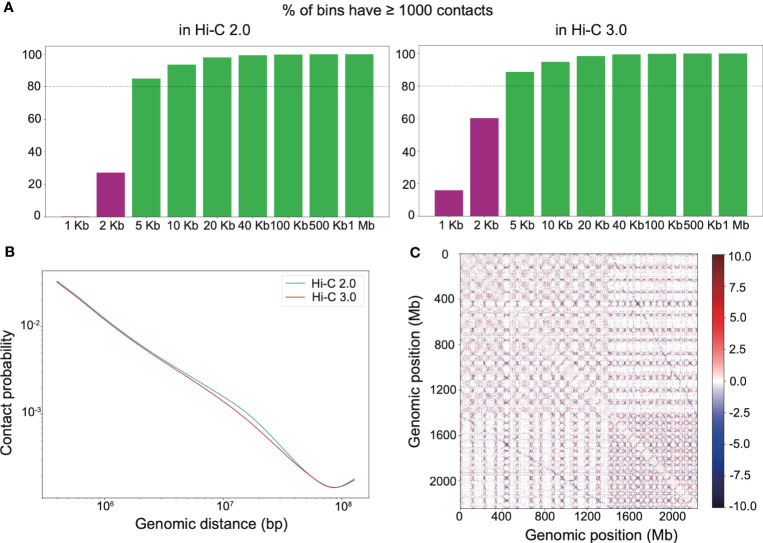
Hi-C 3.0 detects more contacts at higher resolution. **(A)** The histogram showed the distribution of mapped Hi-C reads according to the distance between two ends of each read, that is the percentage of bins having least 1000 contact reads for various bin sizes (resolutions). **(B)** Chromatin contact probability relative to genomic distance at a resolution of 200 kb. **(C)** Whole-genome relative interaction heatmap produced by subtracting Hi-C 2.0 signals from Hi-C 3.0 signals, illustrating their differences at a resolution of 100 kb. Positive values (red) indicate more contacts detected with the Hi-C 3.0 data, negative values (blue) are the opposite. Dotted lines distinguish each chromosome.

Adequate resolution of Hi-C library gives the capacity to detect more delicate chromatin structures, especially loops. It turned out that both Hi-C 2.0 and 3.0 reached the resolution of 5 kb (**Figure 3A**). At smaller resolutions, Hi-C 3.0 produced more bins containing over 1,000 reads than did Hi-C 2.0 (**Figure 3A**). Notably, PCR duplicates may account for up to 20% of valid interactions in general Hi-C libraries. However, there were no duplicate interactions in our Hi-C data (**Supplementary Table S2**), which implied that deeper sequencing of our libraries had the potential to achieve more valid data. Hence, the actual resolution of Hi-C 3.0 may exceed that of Hi-C 2.0 for a given amount of data if adequate sequencing depth was obtained. Chromatin contact probability shows a general negative trend in interaction frequency with increased linear distance. For Hi-C 3.0 data, the contact decay curve had a steeper slope that may attribute to the reduced fragment mobility and the decreased spurious ligations (**Figure 3B**).

Hi-C interaction heatmaps at a resolution of 200 kb showed that the intra-chromosomal contacts were apparently stronger than the inter-chromosomal contacts (**Supplementary Figure S4**). In addition, interaction matrices of different resolutions suggested that Hi-C 3.0 detected more loops than Hi-C 2.0 (**Supplementary Figure S5**). The relative interaction heatmap exhibited blue dots away from and red dots near the matrix diagonal within the chromosome or at the genome-wide level, demonstrating that Hi-C 3.0 obtained fewer long-range or *trans* contacts, while detected more short-range *cis* contacts (**Figure 3C**). Relative interaction heatmaps of individual chromosomes also showed stronger short-range signals in Hi-C 3.0 (**Supplementary Figure S6**). Intriguingly, the relative heatmap displayed blue dots between the homologous chromosomes, indicating Hi-C 3.0 effectively decreased the noise signal of A and D sub-genomes in cotton (**Figure 3C**). It suggested that Hi-C 3.0 can reveal the 3D genomic structures for allopolyploid plants. All told, the results showed that Hi-C 3.0 has the ability to improve the resolution of Hi-C matrices and detect more interactions with fewer misleading ligations.

### 3.5 Hi-C 3.0 strengthens the detection of loops

Chromatin regions contact with each other through significant interactions when distant DNA sites are close in space. These spatial proximity interactions form chromatin loops that can involve domains with different biological functions (Li et al., 2012; De Laat and Duboule, 2013). Of all structural features, the detection of loops depends most on sequencing depth and quality.

**Figure 4:**
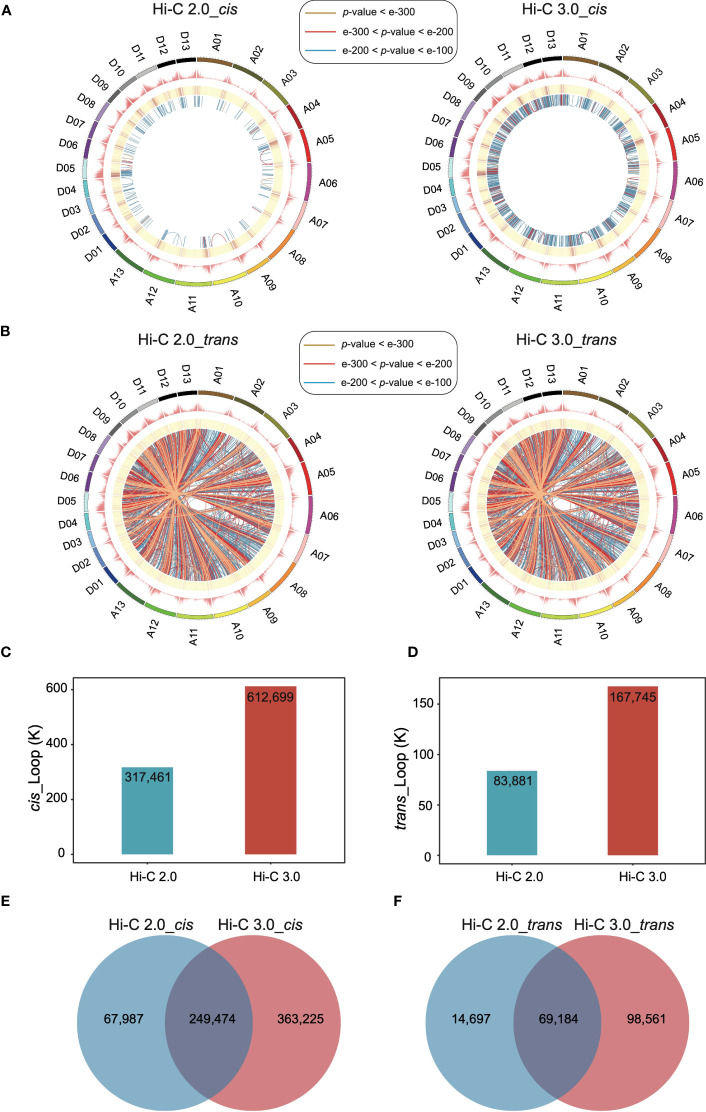
Hi-C 3.0 strengthens the detection of loops. **(A)** The circus plots showed the genome-wide significant intra-chromosomal (*cis*) loops at a resolution of 10 kb detected by Hi-C 2.0 data (left) and Hi-C 3.0 data (right). The circles from outer to inner respectively indicate chromosomes and their locations, number of genes, enrichment of the significant *cis* interaction sites, and links between two *cis*-loop anchors (darker blue represents a smaller *p*-value). The most significant *cis* interactions are displayed in yellow (*p*-value < e-300), red (e-300 < *p*-value < e-200) and blue (e-200 < *p*-value < e-100). **(B)** The circus plots showed the genome-wide significant inter-chromosomal (*trans*) loops at a resolution of 10 kb detected by Hi-C 2.0 data (left) and Hi-C 3.0 data (right). The figure parameters were the same with **(A)**. The histogram showed the total number of the detected *cis*-loops **(C)** and *trans*-loops **(D)** by two protocols. Venn diagram showed the differential detection of *cis*-loops **(E)** and trans-loops **(F)** between Hi-C 2.0 and 3.0.

The extent of interactions between every pair of bins was analyzed to detect loops at a resolution of 10 kb. In Hi-C 3.0 data, the significant *cis* interactions between nonadjacent bins increased a lot (**Figure 4A**), so did the significant *trans* interactions (**Figure 4B**). More significant interactions enhanced the capability of loop detection remarkably. Loops within the same chromosome were categorized as *cis* loops, and those between chromosomes are *trans* loops. Furthermore, Hi-C 3.0 improved the strength of loop signals by obtaining more interactions to support the detection of the same loops with those in Hi-C 2.0 (**Figures 4A, B**). Intriguingly, the distribution of loop anchors showed high correlation with the gene density that occurs at the ends of chromosomes (**Figures 4A, B**). This trend implied the potential of loops associated with the open chromatin regions for active gene expression.

In addition, increased significant interactions of Hi-C 3.0 data resulted in more loops, as much as twice the number detected by Hi-C 2.0 (**Figures 4C, D**). The majority of loops detected based on Hi-C 3.0 overlapped with that based on Hi-C 2.0. However, there was a large part of loops specifically detected by Hi-C 3.0 (**Figures 4E, F**, **Supplementary Figure S7**). Apart from the same loops, Hi-C 3.0 detected more loops of short-range or between the regions of gene and non-gene to better depict the regulation mechanism, showing the superiority of extra cross-linking and finer fragmentation (**Supplementary Figure S7**).

### 3.6 Hi-C 3.0 expands the range of compartment detection

**Figure 5:**
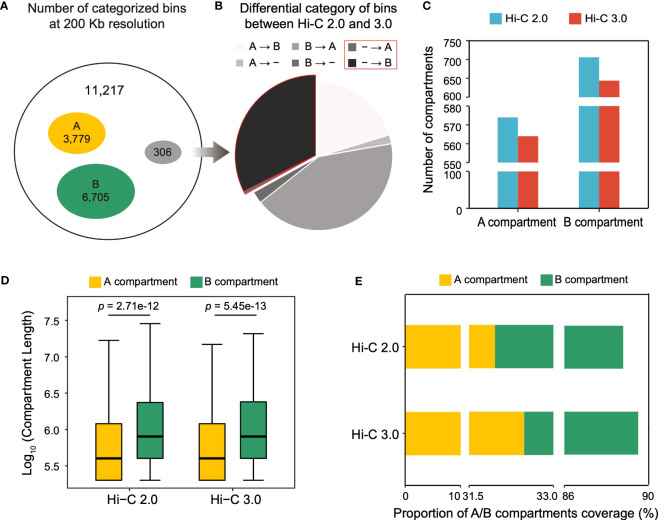
Hi-C 3.0 expands the range of compartment detection. **(A)** At the resolution of 200 kb, concurrence of bin typing between Hi-C 2.0 and 3.0. Total bins (black), both A compartment (yellow), both B compartment (green), and differential classification (grey). **(B)** Breakdown of differentially categorized bins in Figure 5A. A represents A compartment, B represents B compartment, - represents bins without compartment annotation, → represents a switch from Hi-C 2.0 to Hi-C 3.0. **(C)** Histogram showed the number of A/B compartment structures detected by two protocols. **(D)** The box plots showed the length distribution of A/B compartment structures. Significance determined by Wilcoxon rank-sum test. **(E)** Proportions of genomic region covered by A/B compartments.

Compartments are divided into A and B types. A compartment represents open chromatin areas with enrichment of genes and active histone modifications, what is known as euchromatin. Meanwhile, B compartment has the opposite characteristics and is known as heterochromatin. The cotton genome was divided into 11,217 bins at a resolution of 200 kb, of which 3,779 were mutually categorized into type A and 6,705 into type B in both Hi-C data (**Figure 5A**). However, 306 bins were differentially classified, of which 30% bins lacked annotations in Hi-C 2.0 were able to be categorized in Hi-C 3.0, suggesting that Hi-C 3.0 resulted in a higher resolution for the detection of compartment (**Figure 5B**).

The compartment structure is formed by contiguous stretches of bins of the same type. Notably, despite having more bins that belonged to compartments, Hi-C 3.0 identified fewer compartments than Hi-C 2.0 (**Figure 5C**). Therefore, the compartment length was evaluated and revealed two important trends (**Figure 5D**). First, the average length of B compartment was dramatically greater than that of A compartment. Second, the lengths of A/B compartments were higher in Hi-C 3.0 (3,910/6,866 bp) than as determined by Hi-C 2.0 (3,847/6,842 bp), which explained the effect of more bins accounting for fewer compartments in Hi-C 3.0. This implied that Hi-C 3.0 may reduce spurious compartments and determine the ranges of compartments more precisely. It was further supported by the fact that compartment in Hi-C 3.0 covered more genomic region than that in Hi-C 2.0 (**Figure 5E**). Wherein, the coverage of A compartment in Hi-C 3.0 was higher than that in Hi-C 2.0, although it remained the fact that B compartment occupied more area than A compartment in general (**Figure 5E**). Moreover, both Hi-C data indicated that A compartment had denser gene and lower GC content than B compartment (**Supplementary Figure S8**). In conclusion, these results revealed that Hi-C 3.0 contributes to the detection of compartments by expanding the range.

### 3.7 Compartmentalization with Hi-C 3.0 tend to be more reliable

**Figure 6:**
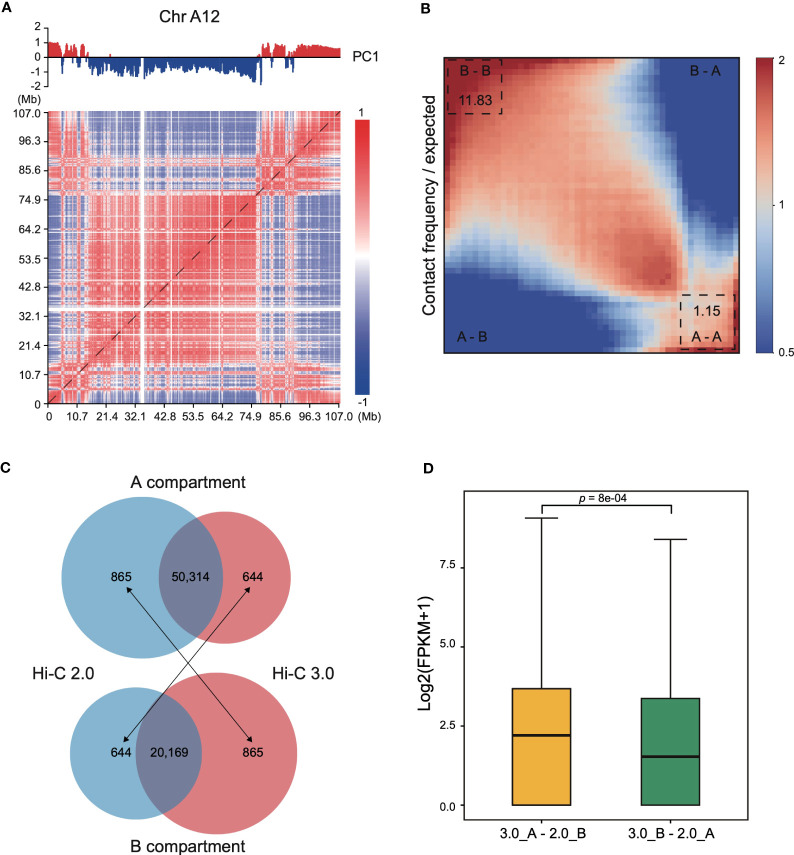
Compartmentalization with Hi-C 3.0 tend to be more reliable. **(A)** Interaction heatmap of chromosome A12 using Hi-C 3.0. The upper tracks showed principal component 1 (PC1) values generated for the genomic segments that are displayed below at a resolution of 200 kb. Positive values (red) represent A compartments and negative values (blue) B compartments. **(B)** Saddle plot of the Hi-C 3.0. A indicates A compartment, B indicates B compartment. The upper left of the matrix represents the strongest B-B interactions, the lower right represents the strongest A-A interactions, and the lower left and upper right represent A-B and B-A, respectively. **(C)** Number of genes categorized into A/B compartments. Black arrows indicate genes differentially annotated between the two protocols. **(D)** Box plot showed the expression levels of genes differentially classified into A/B compartments between Hi-C 2.0 and 3.0 samples in **(C)**. The label 3.0_A-2.0_B represents 644 genes in **(C)** and 3.0_B-2.0_A represents 865 genes in **(C)**. Significance determined by two-sided Wilcoxon signed-rank test.

Interaction heatmap generated from Hi-C 3.0 data showed the distribution of compartments along chromosome arms (**Figure 6A**). A compartment localized to the two ends, while B compartment distributed in the interior around the centromere. The degree of contrast between the domains that comprise the A/B compartments varies between protocols employing different cross-linking strategies or restriction enzymes (Akgol Oksuz et al., 2021). For instance, interaction matrices obtained with a single cross-linker and shorter digestion display a relatively weak compartment pattern, whereas those obtained with additional cross-linking and larger fragments show much stronger patterns. Here, a saddle plot of the genome-wide interaction map revealed that compartments of the same type had a higher frequency of contacts than compartments of different types (**Figure 6B**). The compartment patterns were both strong and exhibited no obvious differences between Hi-C 2.0 and 3.0 data (**Supplementary Figure S9**), except that Hi-C 3.0 resulted in a stronger compartment strength only in preferential B-B contacts. These findings proved that Hi-C 3.0 maintains a good balance between shorter fragmentation and strong compartment pattern.

At last, genes categorized into A/B compartments in Hi-C 2.0 and 3.0 samples were examined with their expression pattern. Most of genes shared the same classification, with 50,314 genes consistently annotated in A compartment and 20,169 genes in B compartment (**Figure 6C**). However, 1,509 genes were differentially categorized, of which 644 genes annotated to A compartment in Hi-C 3.0 but B compartment in Hi-C 2.0, and the other 865 genes annotated to B compartment in Hi-C 3.0 but A compartment in Hi-C 2.0. To validate the reliability of the gene annotation on A/B compartment by Hi-C 2.0 versus Hi-C 3.0, the gene expression activity with transcriptome data of cotton leaf (Zhang et al., 2015) were examined. Overall, the expression level of 644 genes were significantly higher than that of 865 genes (**Figure 6D**). Moreover, these 644 genes predominantly distributed at the ends of chromosome or the gene-rich region, while the 865 genes did not showed the trend (**Supplementary Figure S10**, **Figure S11**). These results suggested that the 644 genes resembled A compartment feature and the 865 genes were more like B compartment. Taken together, genes categorized into A/B compartments with Hi-C 3.0 might be more reliable.

## 4 Discussion

Hi-C technology is employed in various plant species and tissues to reveal the role of chromatin interactions in mediating growth, development, and stress responses (Li et al., 2015; Perrella et al., 2020). However, constructing the high quality Hi-C library remains immensely challenging because of technical barriers (Ouyang et al., 2020a). The continuous evolution of Hi-C technology highlights two key factors that affecting data quality, the cross-linking agent and the chromatin digestion strategy. Here in the presented study, multiple optimizations were successfully applied to the upgraded method using the sample of cotton leaf. Moreover, the modified procedures of Hi-C 3.0 were tested with other plant samples, such as leaves from *Arabidopsis* and soybean in the laboratory. Therefore, we believe that Hi-C 3.0 can be effectively applied in plants by following the detailed protocol step by step.

First of all, distinct cross-linking agents with different lengths of molecular arms lead to diverse distances of fixed space, and directly affect the interaction ranges that can be detected (Akgol Oksuz et al., 2021). For instance, the most common cross-linking chemistry is FA, which links groups that are separated by ~2-Å (Hoffman et al., 2015). Thus, FA is well suited for capturing the interactions of macromolecules in close proximity. Other cross-linking agents have longer molecular arms and so can accomplish cross-linking at longer distances, such as DSG (an 8-Å crosslinker) (Strang et al., 2001) and EGS (a 16.1-Å crosslinker) (Tian et al., 2012; Hsieh et al., 2020). Thus, Hi-C 3.0 applied DSG in addition to FA, the extra cross-linking significantly decreased spurious ligations because of the stronger connection between truly interacting fragments. In particular, the double cross-linking chemistry yielded more intra-chromosomal contacts, which increased the signal-to-noise ratio and improved the efficiency of the generated data. Due to retaining more *in situ* interactions, the detection of loops and compartments was strengthened simultaneously.

Secondly, enzymes digest chromatin into distinct segments of different sizes, which determine the DNA fragmentation status and the final resolution of Hi-C matrices (Su et al., 2021). In general, smaller chromatin fragments yield more short-range interactions at the cost of losing some long-range interactions. It is noteworthy that longer fragmentation could decrease random ligations. Compartment signals are stronger for libraries with longer fragments, while loop identification capability reaches its apex when the chromatin is digested into the extremely small fragments with mNase (Lieberman-Aiden et al., 2009; Hsieh et al., 2015; Belaghzal et al., 2017). Hi-C 3.0 used double restriction endonuclease enzymes (*Dpn*II and *Dde*I), which produced an intermediate fragment length between the conventional Hi-C 2.0 using single enzyme and the Micro-C of nucleosome-sized fragments. Consequently, Hi-C 3.0 was able to obtain more reliable contacts to detect loops and also compensated for the shortcoming of smaller fragments reducing compartmentalization strength. It is possible that compartmental interactions are in general more difficult to capture than loop interactions because looping structures are closely held together by cohesion-like complexes.

In addition to the two strategic steps mentioned above, we optimized the isolation of plant nuclei to decrease background noise and improved the library quality. A recommendation of systematic quality controls as part of the Hi-C 3.0 experimental procedure were also provided. This can help ensure the success of library preparation, especially with wide diversified plant species.

## 5 Conclusions

For research into genome-wide spatial interactions, Hi-C 3.0 is a more efficient choice compared to conventional Hi-C. This is due to its ability to obtain more contact signals from a given amount of data, which can improve data efficiency and reduce sequencing costs. It is recommended to use additional cross-linking chemistry in Hi-C assays. The enzyme selection for fragmentation should depend on the purpose of the study.

## Data availability statement

Novel data generated in this study have been deposited in the Genome Sequence Archive in National Genomics Data Center, China National Center for Bioinformation / Beijing Institute of Genomics, Chinese Academy of Sciences (GSA: CRA011393) that are publicly accessible at https://ngdc.cncb.ac.cn/gsa.

## Author contributions

JH, XG and LF conceptualized the project. JH designed and performed the Hi-C library construction experiments. JH, SW, and TZ conducted the bioinformatic analysis and organized the results. HW assisted in carrying out experiments. JH, XG and LF wrote the manuscript. All authors contributed to the article and approved the submitted version.

## Supplementary material

Supplementary Figure 1 Quality control of key steps in the Hi-C 2.0 protocol. **(A)** Intact primary genomic DNA and the chromatin digested by *Dpn*II. Chromatin after digestion shows a smaller smear size around 1000-3000 bp. **(B)** Adjacent DNA fragments ligated by T4 DNA ligase. Chromatin fragments after ligation show a higher molecular weight on the whole. **(C)** DNA fragmentation to size of ~200-500 bp by sonication. Chromatin fragments after sonication exhibited a lower distribution. **(D)** Evaluation of the final Hi-C 2.0 library before or after fragment selection and purification. **(E)** Digestion of the final Hi-C 2.0 library by *Cla*I. The digested library shows a lower molecular weight. M represented Marker. Ladder bands from top to bottom were 5000, 3000, 2000, 1000, 750, 500, 250, and 100 bp, respectively.

Supplementary Figure 2 Evaluation of the Hi-C 2.0 data *via* the HiC-Pro pipeline. **(A)** Size distribution of valid pairs. **(B)** Quality control of read alignment. **(C)** Histogram showed the distribution of the classified read pairing. Low quality alignments, singletons, and multiple hits are usually removed for subsequent analyses. **(D)** Filtering of read pairs. The fraction of duplicated reads and of short range versus long range interactions were reported. **(E)** Histogram showed the read pairs aligned on restriction fragments. Invalid pairs, such as dangling-end and self-circle, are good indicators of library quality and are tracked but discarded for subsequent analysis. The results shown are from one replicate of the Hi-C 2.0 sample, the other is similar.

Supplementary Figure 3 Schematic chart for the read pair alignments. **(A)** Codes UU (unique-unique), UR (unique-rescued), and RU (rescued-unique, equivalent to UR considering the paired reads) represent valid read pairs. **(B)** Codes NU (null-unique), NN (null-null), MU (multi-unique), MM (multi-multi), NM (null-multi), and WW (walk-walk) represent invalid read pairs. U indicates uniquely mapped reads. R indicates reads that can be considered as unique mapping through rescue. N indicates unmapped reads. M indicates non-specifically mapped reads. W indicates unavailable reads.

Supplementary Figure 4 Interaction heatmaps generated from the Hi-C data. Whole-genome Hi-C interaction heatmaps at the resolution of 200 kb.

Supplementary Figure 5 A representative Hi-C matrices at multi-resolutions. Hi-C matrices of chromosome D05: 0-64 Mb, 10-20 Mb, and 16-18 Mb at resolutions of 200 kb, 40 kb, and 10 kb. Black squares in the interaction map indicate loop anchors detected specifically with the Hi-C 3.0 data.

Supplementary Figure 6 Relative interaction heatmaps generated from the Hi-C data. Relative Hi-C interaction heatmaps of individual chromosomes show differences between the Hi-C 2.0 and 3.0 data (Hi-C 3.0 minus Hi-C 2.0) at a resolution of 20 kb. Chromosomes A06, A08, D02 and D05 were shown as representative examples.

Supplementary Figure 7 Chromatin loops detected from the Hi-C data. Chromatin loops are detected at the resolution of 10 kb and shown by curves linking its anchors. The region (50-52 Mb) of chromosome A01 is presented as an example.

Supplementary Figure 8 Gene density and GC content of each bin attributed to A/B compartments. **(A)** Box plot showed the gene density of each bin attributed to A/B compartments. Significance determined by Wilcoxon rank-sum test, no significant difference between Hi-C 2.0 and 3.0 samples. **(B)** Box plot showed GC content of each bin attributed to A/B compartments. Significance determined by Wilcoxon rank-sum test, no significant difference between Hi-C 2.0 and 3.0 samples.

Supplementary Figure 9 Saddle plot of the Hi-C 2.0 data. Saddle plot generated with the PC1 values obtained from the Hi-C 2.0 data. A indicates A compartment, B indicates B compartment.

Supplementary Figure 10 Density of genes differentially classified into A/B compartments. The circles from outer to inner respectively indicate the density of 3.0_B-2.0_A (865) genes, 3.0_A-2.0_B (644) genes and all genes. The minor interval of chromosome scale is 10 Mb.

Supplementary Figure 11 Distribution of the genes differentially classified into A/B compartments. Genes from the 3.0_B-2.0_A (865) and 3.0_A-2.0_B (644) in on chromosome A06. The scale bar indicates the length of chromosome. The color indicates the gene density.

Supplementary Table 1 Summary of key parameters of Hi-C-based technologies.

Supplementary Table 2 Summary of Hi-C data in this study.

Supplementary Table 3 Summary of the previous Hi-C data in plants.
