# Supplementary figures and images for: An upgraded method of high-throughput chromosome conformation capture (Hi-C 3.0) in cotton (Gossypium spp.)
Source: Front Plant Sci. 2023 Jul 4;14:1223591. doi: 10.3389/fpls.2023.1223591 (PMC10353440; doi:10.3389/fpls.2023.1223591)

**A**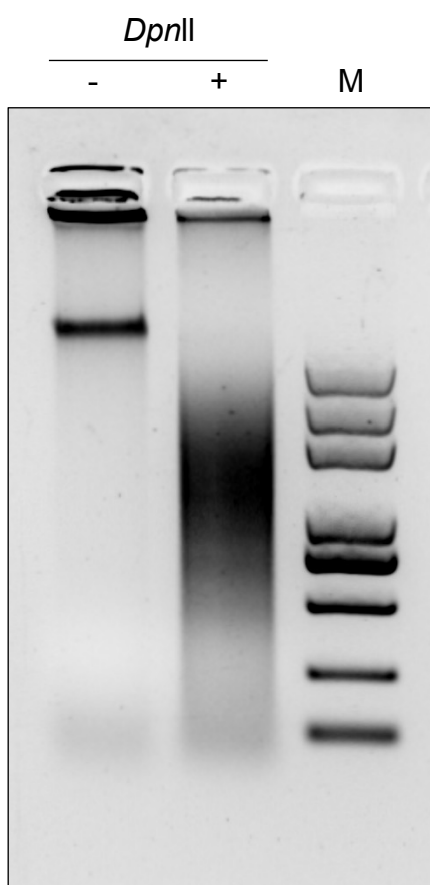**B**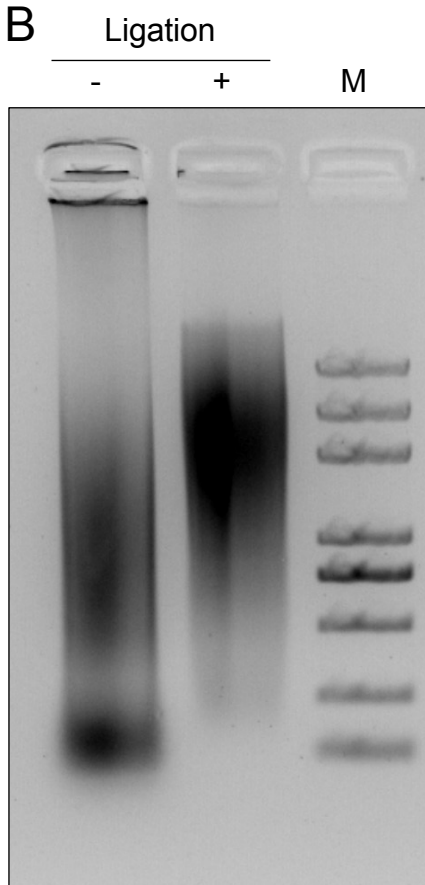**C**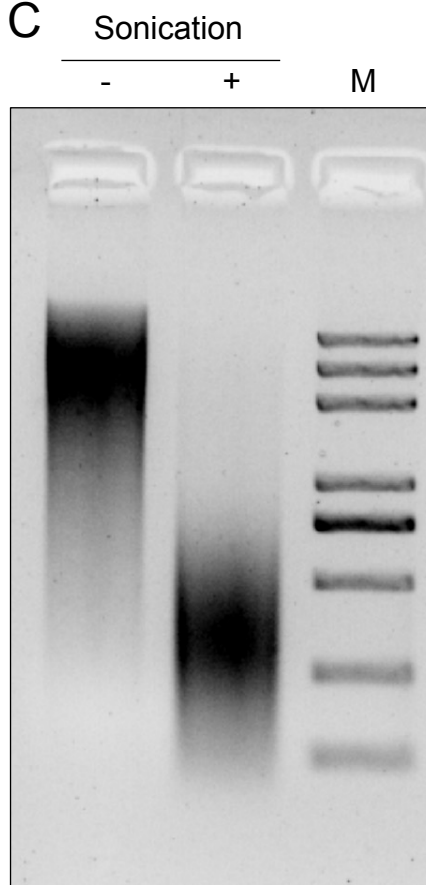**D**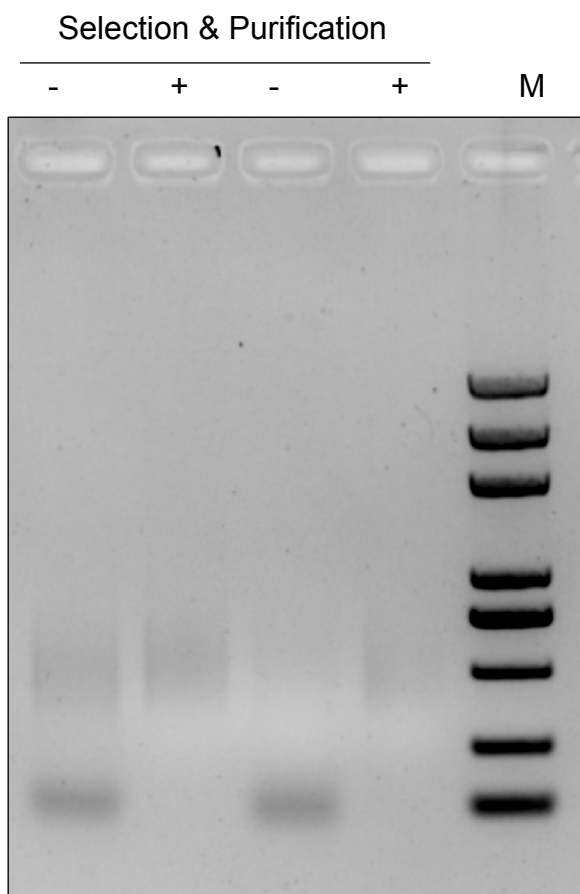**E**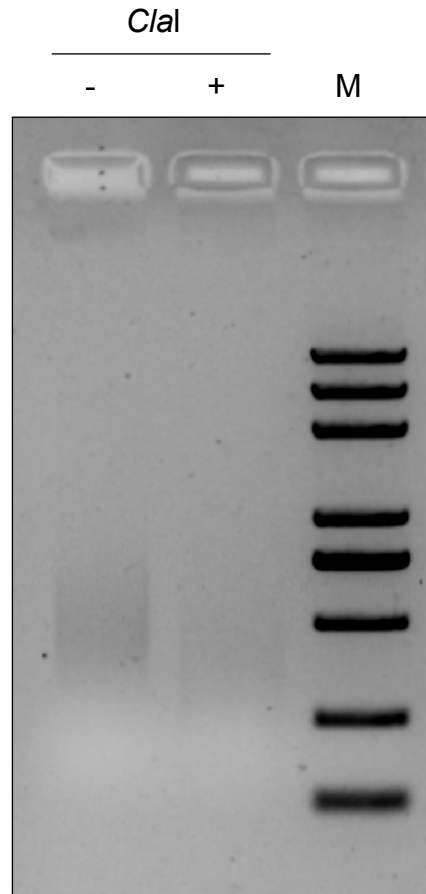

Supplement: Supplementary Figure 1 — Quality control of key steps in the Hi-C 2.0 protocol. (A) Intact primary genomic DNA and the chromatin digested by DpnII. Chromatin after digestion shows a smaller smear size around 1000-3000 bp. (B) Adjacent DNA fragments ligated by T4 DNA ligase. Chromatin fragments after ligation show a higher molecular weight on the whole. (C) DNA fragmentation to size of ~200-500 bp by sonication. Chromatin fragments after sonication exhibited a lower distribution. (D) Evaluation of the final Hi-C 2.0 library before or after fragment selection and purification. (E) Digestion of the final Hi-C 2.0 library by ClaI. The digested library shows a lower molecular weight. M represented Marker. Ladder bands from top to bottom were 5000, 3000, 2000, 1000, 750, 500, 250, and 100 bp, respectively. [file Image_1.pdf]

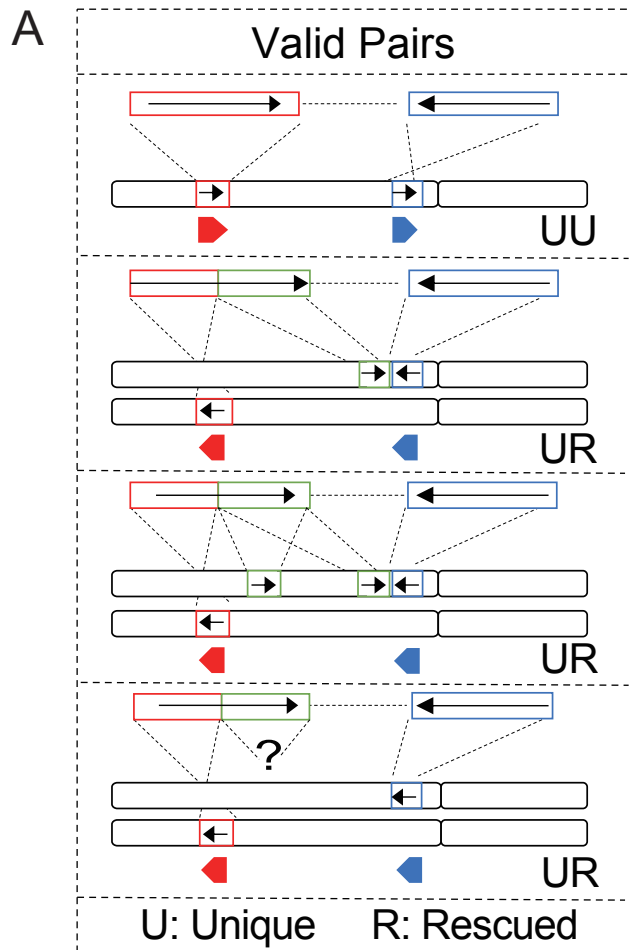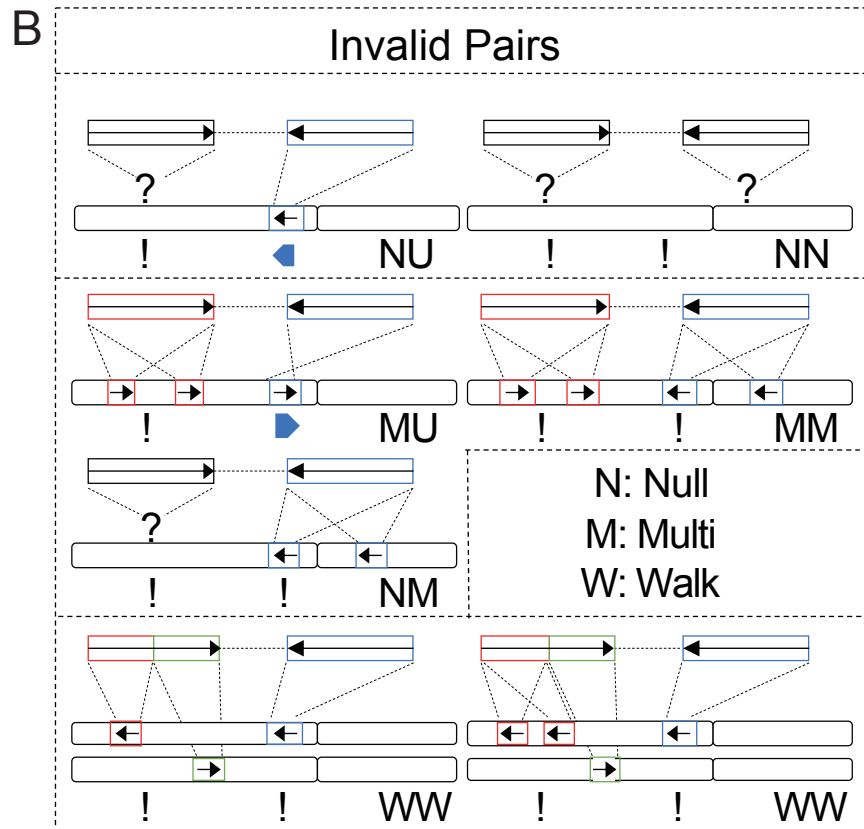

Supplement: Supplementary Figure 3 — Schematic chart for the read pair alignments. (A) Codes UU (unique-unique), UR (unique-rescued), and RU (rescued-unique, equivalent to UR considering the paired reads) represent valid read pairs. (B) Codes NU (null-unique), NN (null-null), MU (multi-unique), MM (multi-multi), NM (null-multi), and WW (walk-walk) represent invalid read pairs. U indicates uniquely mapped reads. R indicates reads that can be considered as unique mapping through rescue. N indicates unmapped reads. M indicates non-specifically mapped reads. W indicates unavailable reads. [file Image_3.pdf]

Hi-C 2.0

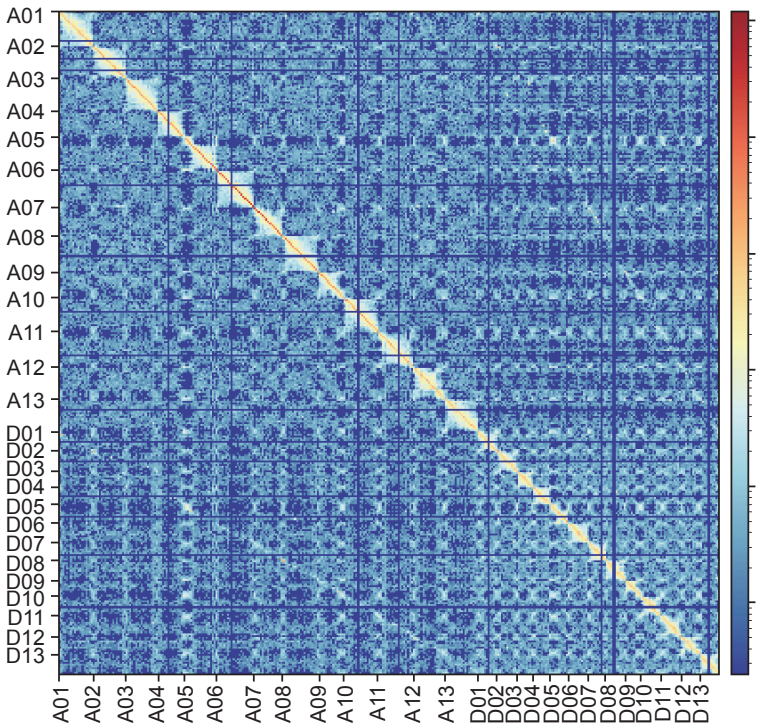

Hi-C 3.0

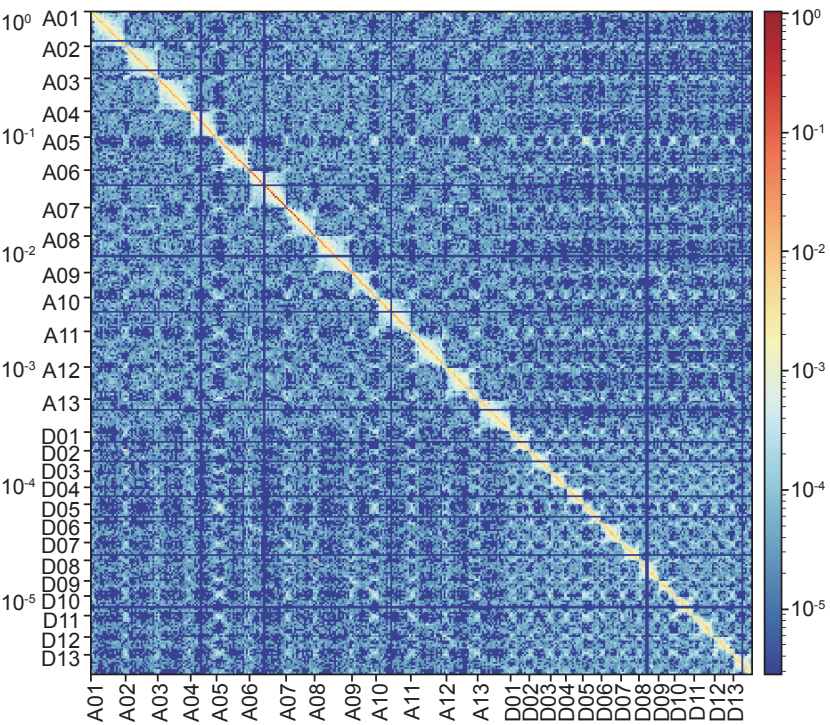

Supplement: Supplementary Figure 4 — Interaction heatmaps generated from the Hi-C data. Whole-genome Hi-C interaction heatmaps at the resolution of 200 kb. [file Image_4.pdf]

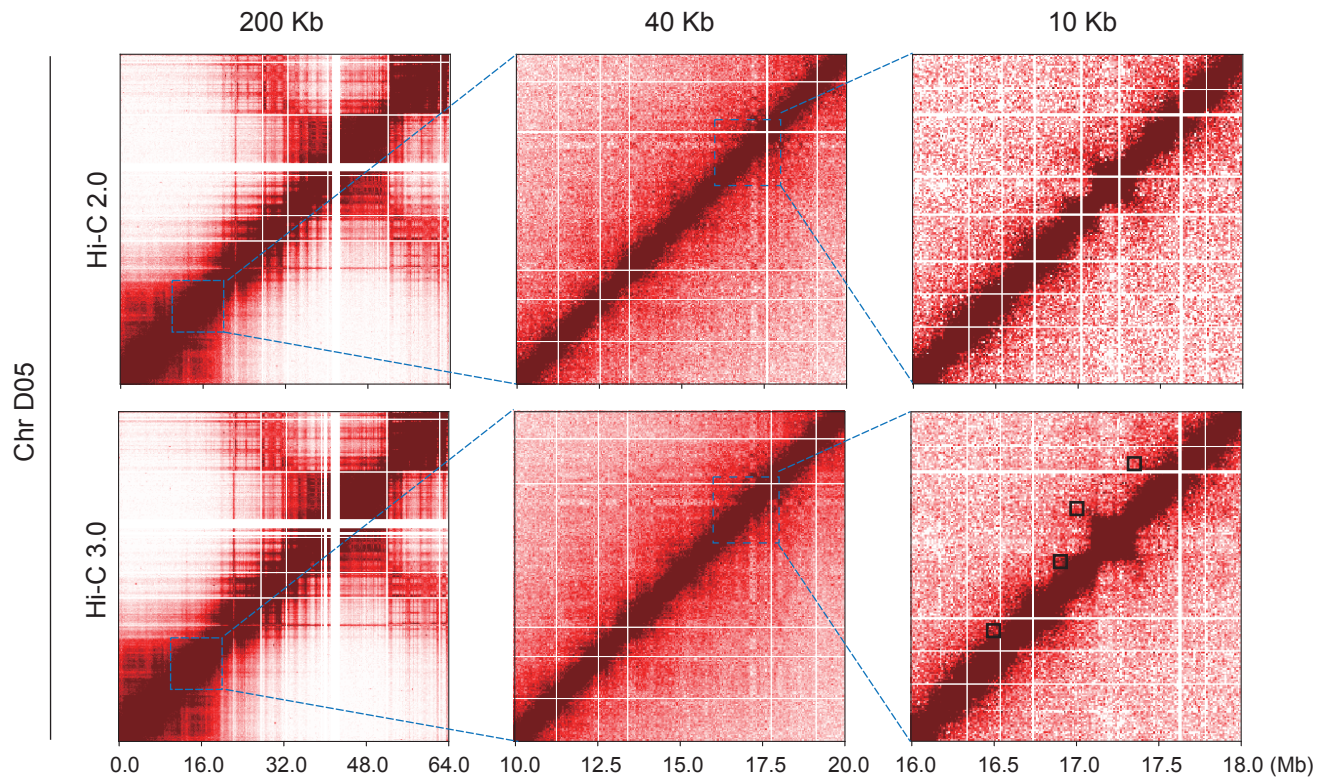

Supplement: Supplementary Figure 5 — A representative Hi-C matrices at multi-resolutions. Hi-C matrices of chromosome D05: 0-64 Mb, 10-20 Mb, and 16-18 Mb at resolutions of 200 kb, 40 kb, and 10 kb. Black squares in the interaction map indicate loop anchors detected specifically with the Hi-C 3.0 data. [file Image_5.pdf]

Chr A06

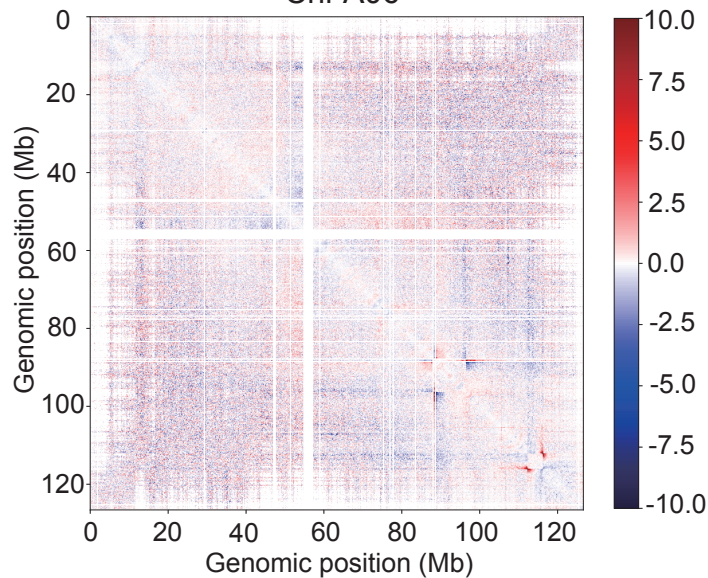

Chr A08

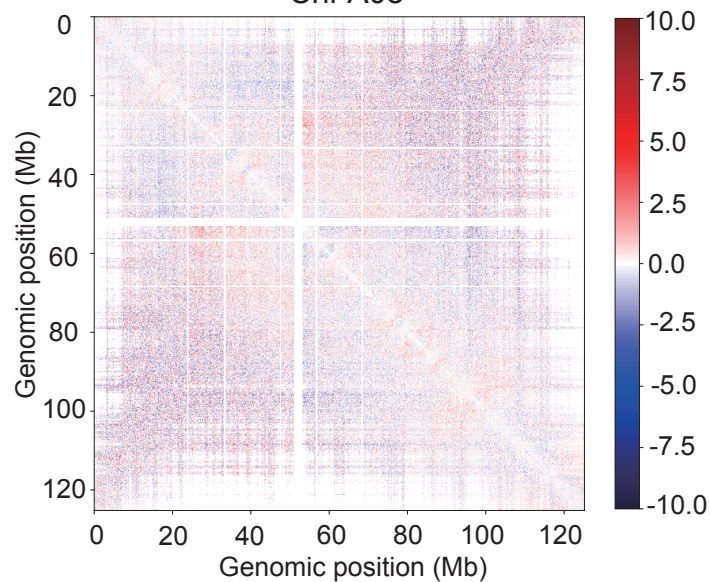

Chr D05

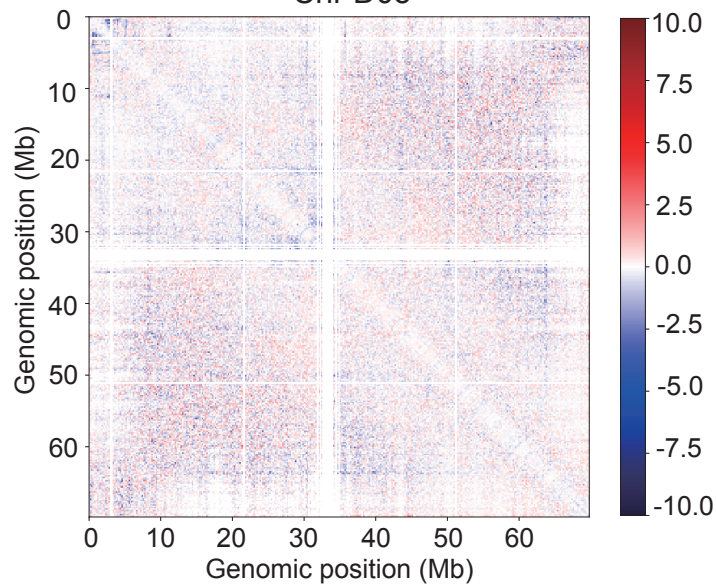

Chr D02

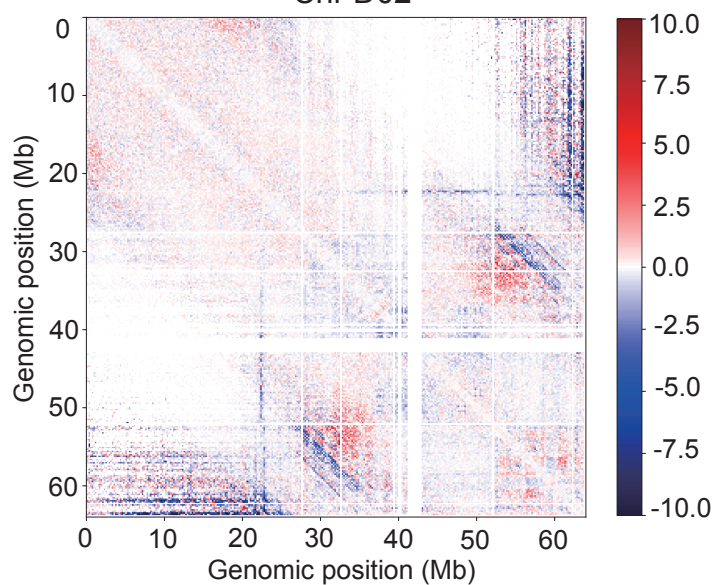

Supplement: Supplementary Figure 6 — Relative interaction heatmaps generated from the Hi-C data. Relative Hi-C interaction heatmaps of individual chromosomes show differences between the Hi-C 2.0 and 3.0 data (Hi-C 3.0 minus Hi-C 2.0) at a resolution of 20 kb. Chromosomes A06, A08, D02 and D05 were shown as representative examples. [file Image_6.pdf]

# Chr A01

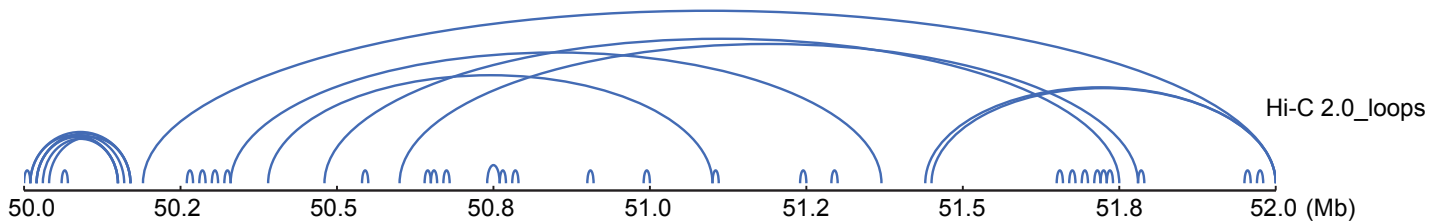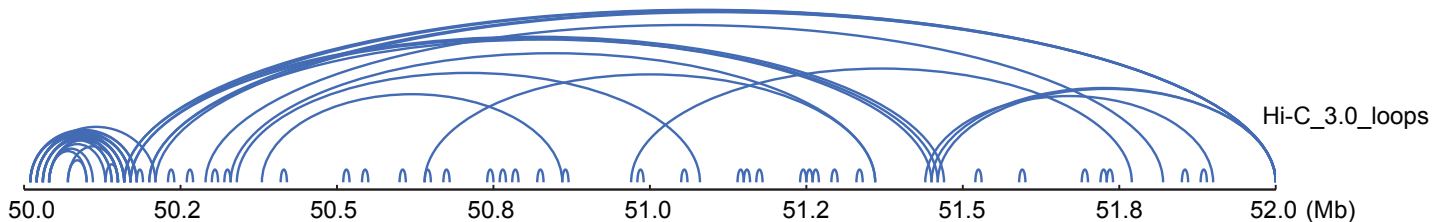

▷GH\_A01G1366

▷GH\_A01G1367  
▷GH\_A01G1368

◀GH\_A01G1369  
◀GH\_A01G1370  
◀GH\_A01G1371  
◀GH\_A01G1372  
◀GH\_A01G1373

GH\_A01G1374 | gene  
GH\_A01G1375 |

Supplement: Supplementary Figure 7 — Chromatin loops detected from the Hi-C data. Chromatin loops are detected at the resolution of 10 kb and shown by curves linking its anchors. The region (50-52 Mb) of chromosome A01 is presented as an example. [file Image_7.pdf]

A

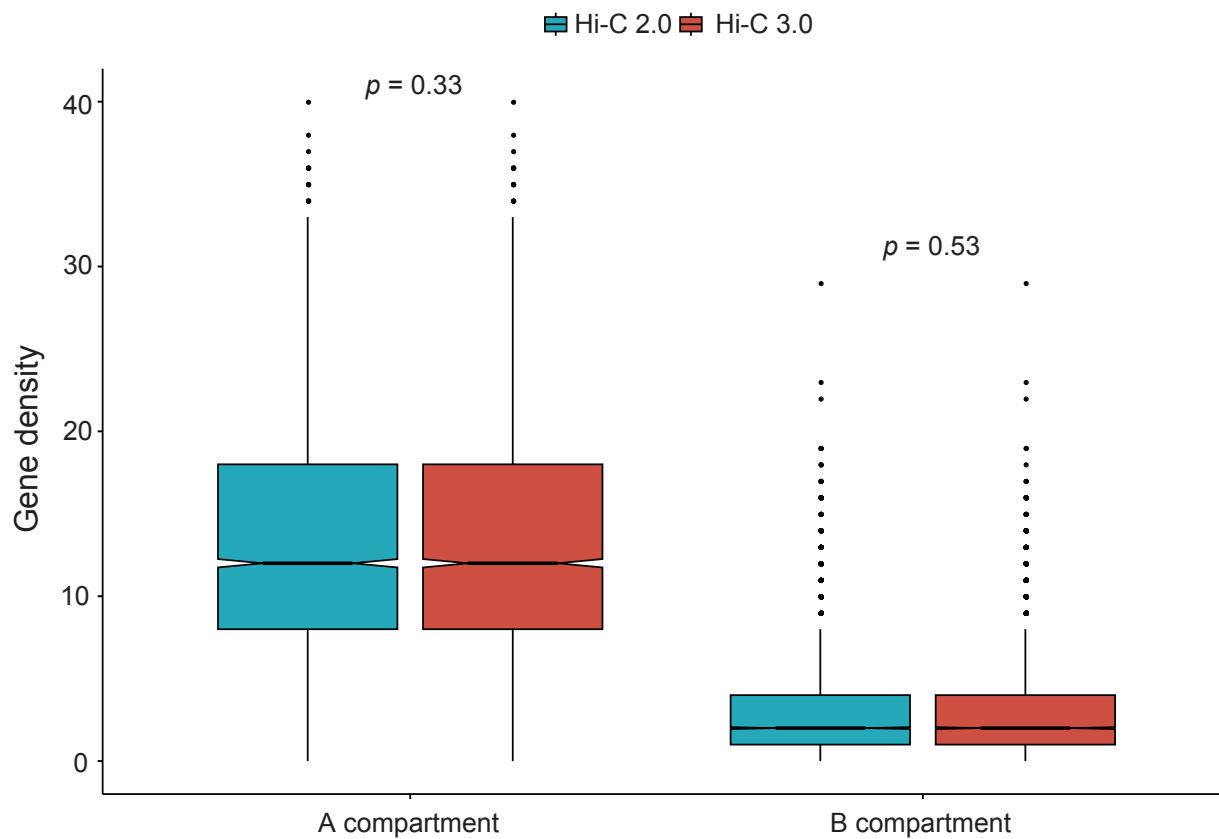

B

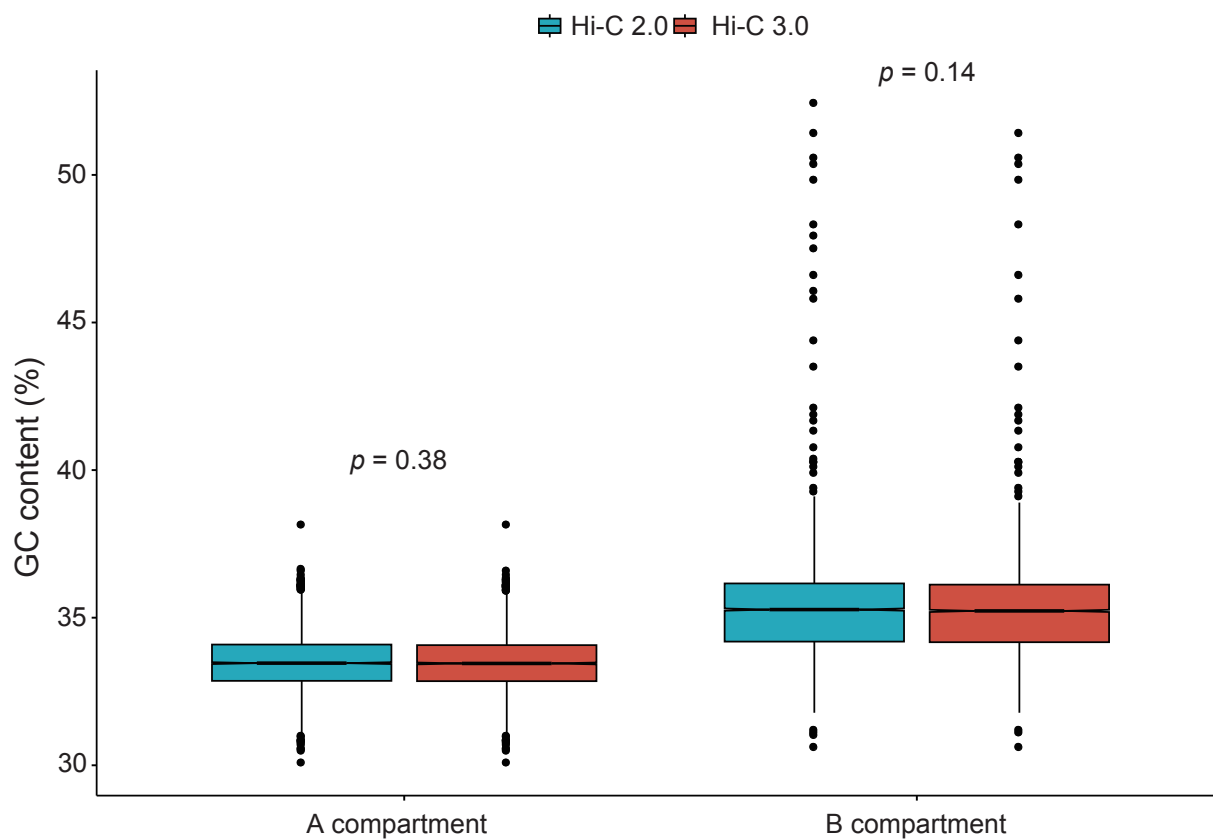

Supplement: Supplementary Figure 8 — Gene density and GC content of each bin attributed to A/B compartments. (A) Box plot showed the gene density of each bin attributed to A/B compartments. Significance determined by Wilcoxon rank-sum test, no significant difference between Hi-C 2.0 and 3.0 samples. (B) Box plot showed GC content of each bin attributed to A/B compartments. Significance determined by Wilcoxon rank-sum test, no significant difference between Hi-C 2.0 and 3.0 samples. [file Image_8.pdf]

contact frequency / expected

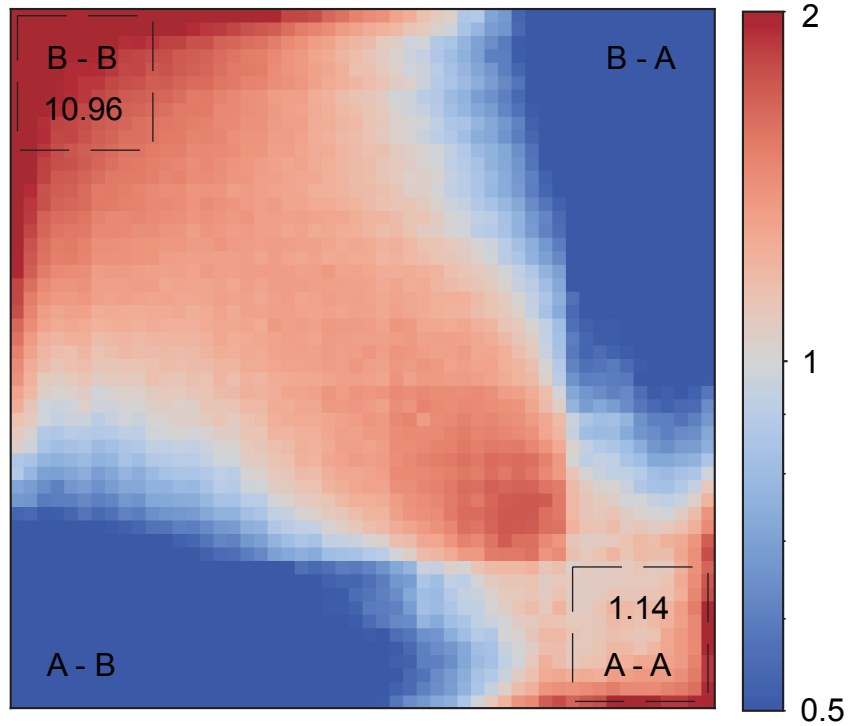

Supplement: Supplementary Figure 9 — Saddle plot of the Hi-C 2.0 data. Saddle plot generated with the PC1 values obtained from the Hi-C 2.0 data. A indicates A compartment, B indicates B compartment. [file Image_9.pdf]

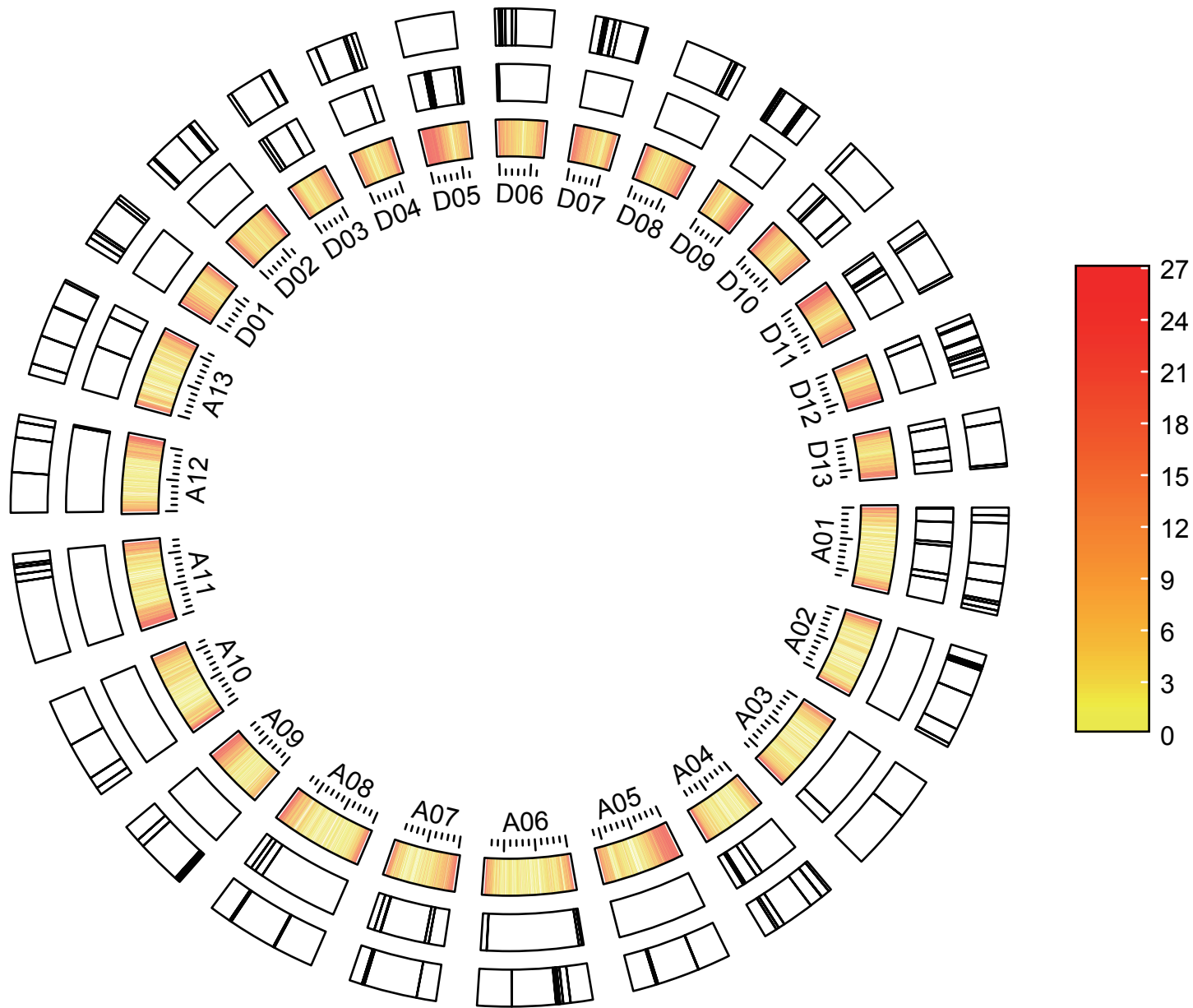

Supplement: Supplementary Figure 10 — Density of genes differentially classified into A/B compartments. The circles from outer to inner respectively indicate the density of 3.0_B-2.0_A (865) genes, 3.0_A-2.0_B (644) genes and all genes. The minor interval of chromosome scale is 10 Mb. [file Image_10.pdf]

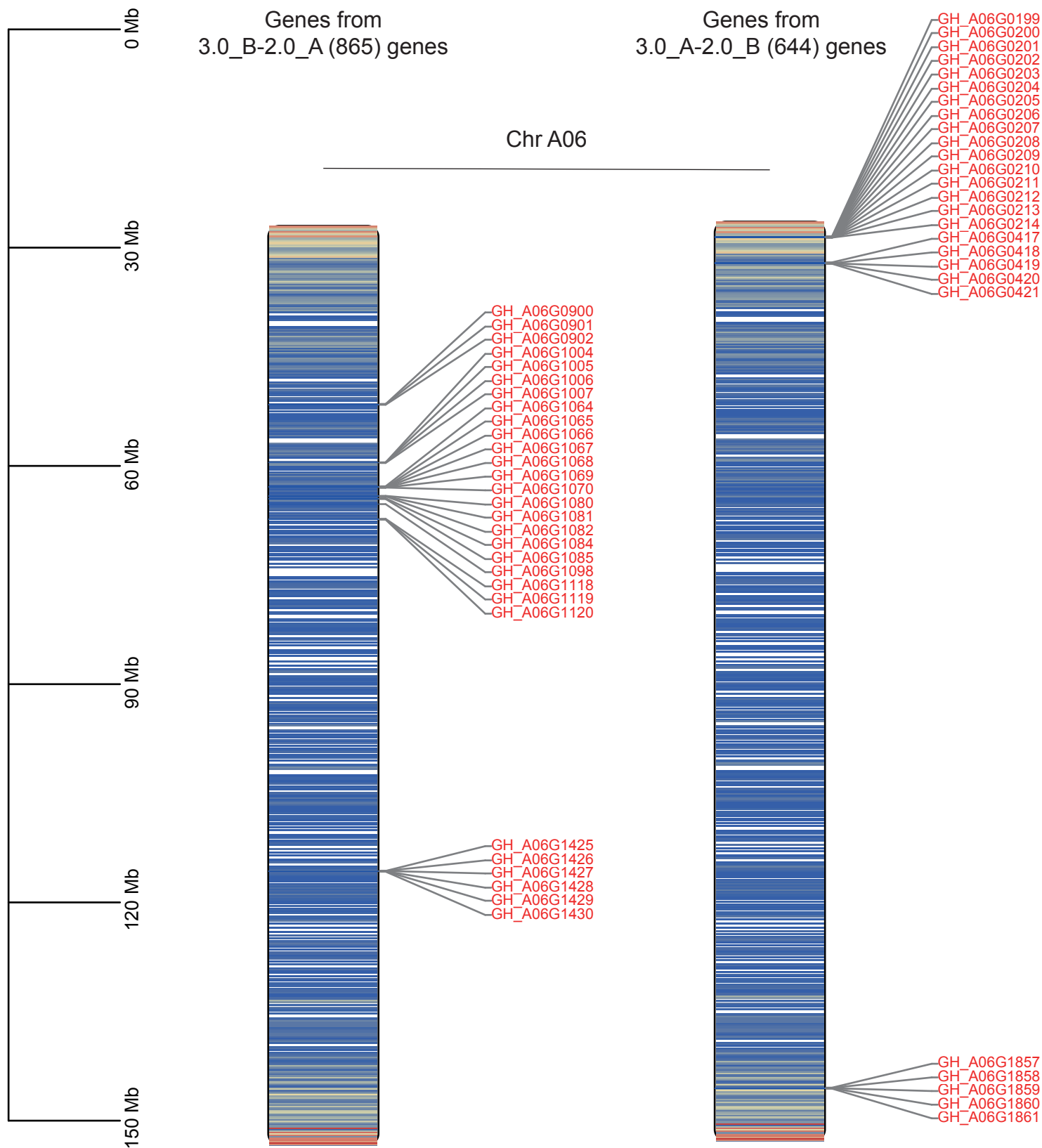

Supplement: Supplementary Figure 11 — Distribution of the genes differentially classified into A/B compartments. Genes from the 3.0_B-2.0_A (865) and 3.0_A-2.0_B (644) in on chromosome A06. The scale bar indicates the length of chromosome. The color indicates the gene density. [file Image_11.pdf]
