# Supplementary material for: An upgraded method of high-throughput chromosome conformation capture (Hi-C 3.0) in cotton (Gossypium spp.)
Source: Front Plant Sci. 2023 Jul 4;14:1223591. doi: 10.3389/fpls.2023.1223591 (PMC10353440; doi:10.3389/fpls.2023.1223591)
Supplement: Supplementary Figure 2 — Evaluation of the Hi-C 2.0 data via the HiC-Pro pipeline. (A) Size distribution of valid pairs. (B) Quality control of read alignment. (C) Histogram showed the distribution of the classified read pairing. Low quality alignments, singletons, and multiple hits are usually removed for subsequent analyses. (D) Filtering of read pairs. The fraction of duplicated reads and of short range versus long range interactions were reported. (E) Histogram showed the read pairs aligned on restriction fragments. Invalid pairs, such as dangling-end and self-circle, are good indicators of library quality and are tracked but discarded for subsequent analysis. The results shown are from one replicate of the Hi-C 2.0 sample, the other is similar. [file Image_2.pdf]

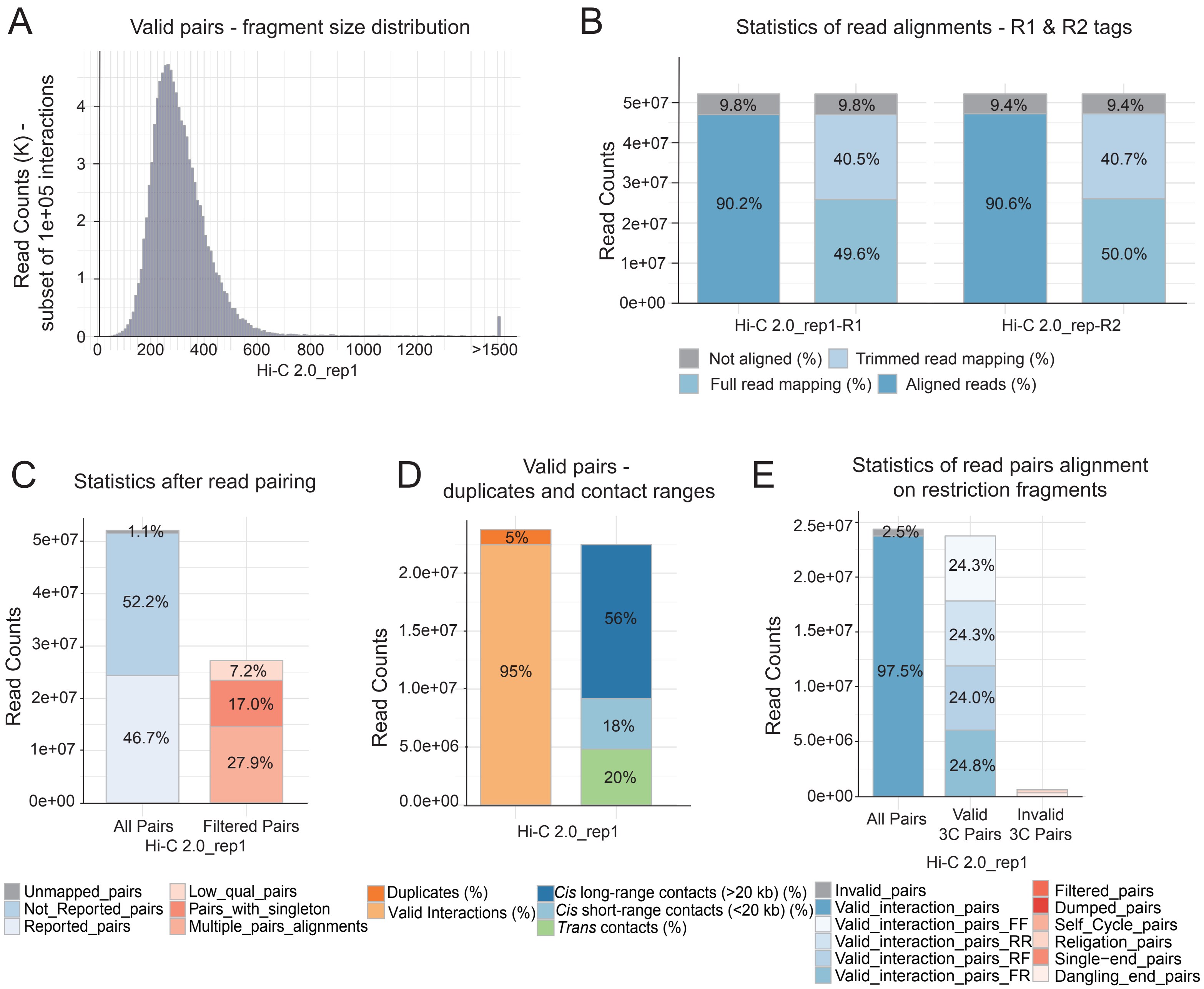
A
Valid pairs - fragment size distribution
Read Counts (K) - subset of 1e+05 interactions
Hi-C 2.0_rep1
0
200
400
600
800
1000
1200
>1500
B
Statistics of read alignments - R1 & R2 tags
Read Counts
9.8%
9.8%
9.4%
9.4%
90.2%
40.5%
49.6%
90.6%
40.7%
50.0%
Hi-C 2.0_rep1-R1
Hi-C 2.0_rep-R2
Not aligned (%)
Trimmed read mapping (%)
Full read mapping (%)
Aligned reads (%)
C
Statistics after read pairing
Read Counts
1.1%
52.2%
46.7%
7.2%
17.0%
27.9%
All Pairs
Filtered Pairs
Hi-C 2.0_rep1
D
Valid pairs - duplicates and contact ranges
Read Counts
5%
95%
56%
18%
20%
Hi-C 2.0_rep1
E
Statistics of read pairs alignment on restriction fragments
Read Counts
2.5%
97.5%
24.3%
24.3%
24.0%
24.8%
All Pairs
Valid 3C Pairs
Invalid 3C Pairs
Hi-C 2.0_rep1
Unmapped_pairs
Not_Reported_pairs
Reported_pairs
Low_qual_pairs
Pairs_with_singleton
Multiple_pairs_alignments
Duplicates (%)
Valid Interactions (%)
Cis long-range contacts (>20 kb) (%)
Cis short-range contacts (<20 kb) (%)
Trans contacts (%)
Invalid_pairs
Valid_interaction_pairs
Valid_interaction_pairs_FF
Valid_interaction_pairs_RR
Valid_interaction_pairs_RF
Valid_interaction_pairs_FR
Filtered_pairs
Dumped_pairs
Self_Cycle_pairs
Religation_pairs
Single−end_pairs
Dangling_end_pairs
